# Pre-Human Immunodeficiency Virus (HIV) infection Th17 CD4^+^ T cells as predictors of early HIV disease progression

**DOI:** 10.1371/journal.ppat.1013852

**Published:** 2026-04-24

**Authors:** Tosin E. Omole, Huong Mai Nguyen, Agata Marcinow, Naima Jahan, Giulia Severini, Nivashnee Naicker, Katherine Thomas, Connie Celum, Nelly Mugo, Andrew Mujugira, James Kublin, Lawrence Corey, Aida Sivro, Jairam Lingappa, Glenda Gray, Lyle R. McKinnon

**Affiliations:** 1 Department of Medical Microbiology and Infectious Diseases, University of Manitoba, Winnipeg, Manitoba, Canada; 2 Vaccine and Infectious Disease Organization (VIDO), Saskatoon, Saskatchewan, Canada; 3 Centre for the AIDS Program of Research in South Africa (CAPRISA), Durban, KwaZulu-Natal, South Africa; 4 Department of Global Health, University of Washington, Seattle, Washington, United States of America; 5 Sexual Reproductive and Adolescent Child Health Research Program, Kenya Medical Research Institute, Nairobi, Kenya; 6 Infectious Diseases Institute, Makerere University, Kampala, Uganda; 7 HIV Vaccine Trials Network (HVTN), Seattle, Washington, United States of America; 8 Vaccine and Infectious Disease Division, Fred Hutchinson Cancer Center, Seattle, Washington, United States of America; 9 National Microbiology Laboratory Branch, Public Health Agency of Canada, Winnipeg, Manitoba, Canada; 10 Department of Medical Microbiology, University of KwaZulu-Natal, Durban, KwaZulu-Natal, South Africa; 11 Departments of Medicine and Pediatrics, University of Washington, Seattle, Washington, United States of America; 12 Infectious Disease and Oncology Research Institute, Faculty of Health Sciences, University of the Witwatersrand, Johannesburg, South Africa; 13 Department of Medical Microbiology and Immunology, University of Nairobi, Nairobi, Kenya; Emory University School of Medicine, UNITED STATES OF AMERICA

## Abstract

Interleukin-17-producing T helper (Th17) CD4^+^ T cells are highly susceptible to HIV infection and are depleted early in people living with HIV. Here, we investigated whether systemic Th17 cell levels prior to HIV infection are associated with subsequent HIV disease progression. We analyzed archived cryopreserved peripheral blood mononuclear cells (PBMCs) collected within one year prior to HIV acquisition from participants enrolled in a South African cohort (HIV Vaccine Trials Network [HVTN] 503; n = 35) and an East African cohort (Partners Pre-exposure Prophylaxis/Couples’ Observational Study [PP/COS]; n = 32). Th17 cell frequencies were quantified by flow cytometry. In HVTN 503, higher pre-HIV IL-17^+^ CD4^+^ T cell frequencies were inversely correlated with CD4/CD8 ratio measured both within 180 days (Spearman rank r_s_ = -0.42, *p* = 0.012) and ≥180 days (r_s_ = -0.55, *p* = 0.001) after HIV infection, and were associated with faster CD4^+^ T cells decline (adjusted hazard ratio [aHR] = 3.5, 95% CI: 1.2 – 9.9, *p* = 0.020). In contrast, no significant association with CD4 decline was observed in the PP/COS cohort (HR = 1.2, 95% CI: 0.4 – 3.4, *p* = 0.795). Sex-stratified analyses in HVTN 503 indicated a more pronounced association between pre-HIV IL-17^+^ CD4^+^ T cells and faster CD4 decline in males than females. In analyses combining all cohorts, higher pre-HIV IL-17^+^ CD4^+^ T cell frequencies remained associated with faster CD4 decline, particularly among younger participants (HR = 3.5; 95% CI: 1.35 – 9.22, *p* = 0.010). Pre-HIV IL-17^+^ CD4^+^ T cell frequencies were not associated with peak or set-point viral load in either cohort. Together, these findings suggest that pre-HIV Th17 cells abundance may influence subsequent HIV disease progression independently of early viral replication.

## Author summary

HIV infection leads to progressive damage of the immune system, but the rate at which this damage occurs varies widely between individuals. Understanding the factors that influence HIV disease progression is essential for improving prevention and treatment strategies. Previous studies have suggested that immune characteristics present before infection may shape disease outcomes after HIV acquisition. In this study, we examined whether the abundance of IL-17-producing CD4^+^ T cells, known as Th17 cells, measured prior to HIV acquisition, were associated with markers of disease progression after infection. We analyzed blood samples from individuals who were HIV-negative at the time of sampling and later acquired HIV during follow-up. We found that individuals with higher pre-infection levels of Th17 cells experienced faster immune decline after HIV infection, including more rapid loss of CD4^+^ T cells and lower CD4/CD8 ratios. These associations were observed independently of viral load and varied by cohort, age and sex. Our findings indicate that immune conditions present before HIV infection can influence subsequent disease progression and suggest that Th17 levels may serve as a potential biomarker to identify individuals at higher risk of rapid HIV-related immune damage.

## Introduction

HIV disease progression is heterogenous, and early events following HIV acquisition are important determinants of HIV reservoir formation and long-term immune parameters. The host immune profile near the time of HIV infection can shape early viral replication and the associated immune damage, which are key predictors of HIV pathogenesis [1–4]. Previous studies have shown that the abundance of HIV target cells prior to infection may accelerate disease progression [1,4,5]. Pre-HIV immune profiles characterized by high expression of the HIV-susceptible migratory marker integrin α4β7 [1,5,6], elevated immune activation markers [3] and increased plasma cytokines [4,7] have been associated with heightened HIV susceptibility and/or faster disease progression post-infection.

A subset of CD4^+^ T cells, Th17 cells, are important targets for HIV and play a crucial role in mucosal immunity, defending against bacterial and fungal infections [8–10]. Th17 cells are enriched at mucosal surfaces, where they maintain barrier integrity and produce pro-inflammatory cytokines, including interleukin-17 (IL-17). [9,10]. IL-17 mediates key host immune mechanisms such as neutrophil recruitment, regulation of mucosal epithelial permeability, and induction of antimicrobial peptides [11,12].

The gastrointestinal mucosa, a primary site for HIV replication, is highly enriched for Th17 cells [13,14]. HIV preferentially infects CD4^+^ T cells, and untreated infection is characterized by progressive CD4^+^ T cell loss, particularly in mucosal tissues [13,14]. Th17 cells are highly permissive to HIV infection and are among the first cells to be depleted during acute infection [15–17], likely due to their mucosal location and high expression of HIV coreceptors CCR5 and CXCR4 [18–20]. Early Th17 depletion contributes to mucosal barrier disruption, increased microbial translocation, and systemic immune activation, which in turn drives HIV disease progression [7,12,17,21,22]. Notably, this rapid Th17 depletion is observed in HIV-susceptible hosts such as humans and rhesus macaques, but not in natural hosts like sooty mangabeys or African green monkeys, nor in HIV-infected long-term non-progressors, who exhibit slower progression to AIDS [17].

While Th17 cells are enriched in mucosal tissues and exhibit distinct functional properties at these sites, they are not restricted to the mucosa and can also be quantified in peripheral blood [23,24]. Circulating Th17 cells represent a dynamic pool of cells that traffic between blood and tissues and include populations expressing mucosal homing receptors such as α4β7 and CCR6, as well as HIV coreceptor CCR5 [19,25,26]. As a result, Th17 cell frequency in blood may reflect broader systemic immune features relevant to mucosal immunity, even though their abundance and function may differ between compartments [23,24]. Importantly, in retrospective longitudinal human cohort studies, peripheral blood provides an accessible means of examining pre-infection immune status. Thus, measurement of Th17 cells in blood provides a practical approach to evaluating baseline immune composition that may influence subsequent HIV disease progression.

Although Th17 cells are recognized as key targets of HIV and their depletion is linked to disease progression, it remains unclear whether the abundance of these cells in peripheral blood before infection influences subsequent HIV outcomes. In this study, we aimed to determine whether higher pre-HIV Th17 cell frequency predicts faster disease progression across multiple African cohorts.

## Results

### Study participants characteristics

We analyzed peripheral blood mononuclear cells (PBMC) samples from individuals enrolled in the HIV Vaccine Trials Network study (HVTN 503, n = 35) and from the Partners Pre-exposure Prophylaxis and Couples Observational Study cohorts (PP/COS, n = 32). All participants were HIV-seronegative at the time of blood draw but subsequently acquired HIV during longitudinal follow-up.

Participants in HVTN 503 were younger, with a median age of 23 years (Interquartile range [IQR]: 22 – 27) compared with those in PP/COS, who had a median age of 30 years (IQR: 25 – 40). The HVTN 503 cohort included 17 female and 18 male participants, while PP/COS included 19 female and 13 male participants. The median time from sample collection to estimated infection was 177 days (IQR: 136 – 219) for HVTN 503 and 225 days (IQR: 116 – 328) for PP/COS. None of the HVTN 503 participants completed the originally planned vaccination regimen, as the trial was stopped early for futility [27]. We use the acronym “PP/COS” to denote participants combined from two concurrent clinical trials involving heterosexual serodiscordant couples: Partners PrEP (n = 25) and COS (n = 7). More than 50% of PP/COS participants had a baseline risk score ≥5, reflecting a population at higher risk of HIV transmission. Baseline characteristics of all participants are summarized in Table 1.

**Table 1:** Baseline characteristics of participants.

Variables	Cohorts	
	**HVTN 503 (*n* = 35)**	**PP/COS (*n* = 32)**
**Age (years)** ^**a**^	23.00 (22.00 – 27.00)	30.00 (25.00 – 40.00)
**Estimated sampling to infection (days)** ^**a**^	177.00 (136.00 – 219.00)	225.00 (116.00 – 328.00)
**CD4 Count(/mm**^**3**^)^**a**^	392.50 (290.00 – 482.50)	497.00 (428.50 – 638.50)
**Sex**		
Female	17 (48.57%)	19 (59.38%)
Male	18 (51.43%)	13 (40.62%)
**Cohort**		
HVTN 503	35 (100%)	NA
Partners PrEP	NA	25 (78.13%)
COS	NA	7 (21.87%)
**Treatment Arm**		
Placebo	17 (48.57%)	14 (43.75%)
Vaccine	18 (51.43%)	N/A
PrEP	N/A	11 (34.38%)
**HSV-2 Status**		
Negative	15 (42.86%)	4 (12.50%)
Positive	20 (57.14%)	17 (53.13%)
**Circumcision (Male)**	6 (17.14%)	0 (0.00%)
**Treatment Status (Off treatment early)**	35 (100.00%)	N/A
**Vaccine Dose Received**		
1	7 (20.00%)	N/A
2	28 (80.00%)	N/A
**AD5 Titre at Baseline**		
≤ 200	12 (34.29%)	N/A
> 200	23 (65.71%)	N/A
**Baseline Risk score**		
< 5	N/A	13 (40.62%)
≥ 5	N/A	19 (59.38%)
**Sites: South Africa**		
Cape town	9 (25.71%)	N/A
eThekwini	7 (20.00%)	N/A
Klerksdorp	6 (17.14%)	N/A
Medunsa	4 (11.43%)	N/A
Soweto	9 (25.71%)	N/A
PHRU	N/A	2 (6.25%)
**Sites: Uganda**		
Jinja	N/A	6 (18.75%)
Kampala	N/A	13 (40.63%)
**Sites: Kenya**		
Kisumu	N/A	4 (12.50%)
Thika	N/A	2 (6.25%)
Nairobi	N/A	5 (15.62%)

Data are presented as sample sizes (n) and corresponding column percentages (%) unless otherwise stated. ^**a**^ Data are presented as Median (Interquartile Range). Abbreviations: HVTN = HIV Vaccine Trials Network; PP/COS = Partners PrEP/Couples Observational Study; PrEP = Pre-exposure Prophylaxis; HSV-2 = Herpes Simplex Virus-2; AD5 = Adenovirus Type 5; PHRU = Perinatal HIV Research Unit (Soweto, South Africa); N/A = Not Applicable.

### Pre-HIV IL-17^+^ CD4^+^ T cells inversely correlate with CD4/CD8 ratio after HIV infection

**Fig 1:**
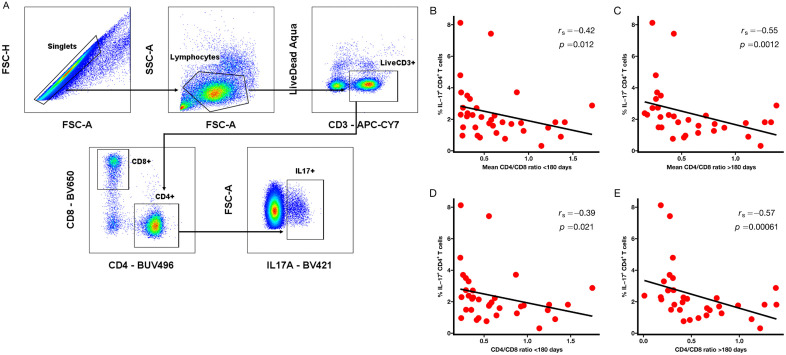
Total pre-HIV IL-17-producing CD4^+^ T cells were correlated with CD4/CD8 ratio in HVTN 503. PBMCs from study participants were stimulated ex vivo for 4 hours with PMA and ionomycin in the presence of Golgi Plug and Golgi Stop. The frequency of CD4^+^ T cells producing IL-17 was measured by flow cytometry. **(A)** Representative flow cytometry gating strategy for total IL-17-producing CD4^+^ T cells. **(B, C)** Correlation with mean CD4/CD8 ratio within the first 180 days post-infection and ≥180 days post-infection. **(D, E)** Correlation with CD4/CD8 ratio at last available measurement within the first 180 days post-infection and ≥180 days post-infection. CD4/CD8 ratios were calculated from absolute CD4 and CD8 counts obtained within the first 180 days (n = 35) or ≥180 days (n = 32) post-infection, as indicated. Measurements obtained after ART initiation or beyond 1-year post-infection were excluded. CD4/CD8 ratio at last available measurement refers to the last CD4/CD8 measurement within the first year of infection prior to ART initiation, if applicable. Correlations were assessed using Spearman rank correlation coefficient **(r**_**s**_), with linear regression lines shown for visualization purposes only. Two-tailed p-values are shown; statistical significance was defined as *p* < 0.05.

We examined the association between pre-HIV Th17 cells, defined as IL-17-producing CD4^+^ T cells following mitogen stimulation (Figs 1A and S1 Fig) and post-infection CD4/CD8 ratios in the HVTN 503 cohort. Mean CD4/CD8 ratios were calculated separately for measurements within the first 180 days following infection and from 180 days until the last measurement prior to ART initiation, if applicable (Fig 1B and 1C). Correlations were additionally examined using each participant’s last available CD4/CD8 ratio measured within the first year of infection, prior to ART initiation (Fig 1D and 1E).

**Fig 2:**
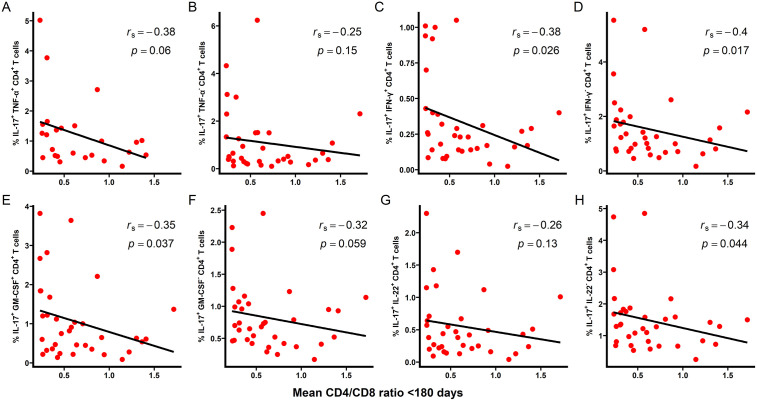
Correlation between pre-HIV IL-17-producing CD4^+^ T cell subsets and mean CD4/CD8 ratio <180 days post-infection in HVTN 503. PBMCs from study participants were stimulated ex vivo for 4 hours with PMA and ionomycin in the presence of Golgi Plug and Golgi Stop. The frequency of CD4^+^ T cells producing IL-17 was measured by flow cytometry. **(A)** IL-17^+^ TNF-α^+^. **(B)** IL-17^+^ TNF-α^-^. **(C)** IL-17^+^ IFN-γ^+^. **(D)** IL-17^+^ IFN-γ^-^. **(E)** IL-17^+^ GM-CSF^+^. **(F)** IL-17^+^ GM-CSF^-^. **(G)** IL-17^+^ IL-22^+^. **(H)** IL-17^+^ IL-22^-^. CD4/CD8 ratios were calculated from absolute CD4 and CD8 counts obtained within the first 180 days (n = 35) post-infection. Measurements obtained after ART initiation or beyond 1-year post-infection were excluded. Correlations were assessed using Spearman rank correlation coefficient **(r**_**s**_), with linear regression lines shown for visualization purposes only. Two-tailed p-values are shown; statistical significance was defined as *p* < 0.05.

**Fig 3:**
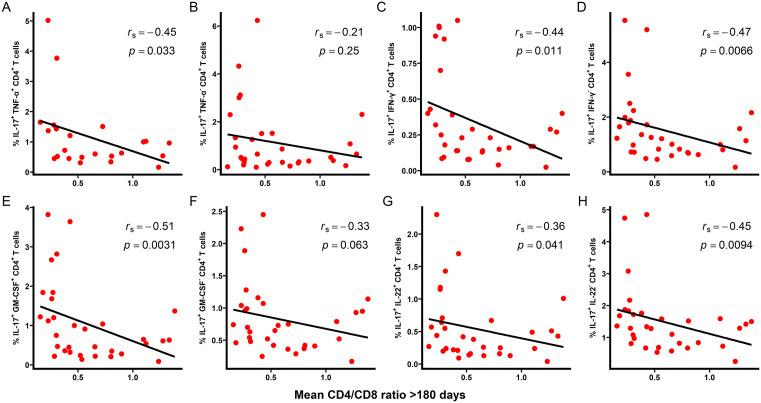
Correlation between pre-HIV IL-17-producing CD4^+^ T cell subsets and mean CD4/CD8 ratio ≥ 180 days post-infection in HVTN 503. PBMCs from study participants were stimulated ex vivo for 4 hours with PMA and ionomycin in the presence of Golgi Plug and Golgi Stop. The frequency of CD4^+^ T cells producing IL-17 was measured by flow cytometry. **(A)** IL-17^+^ TNF-α^+^. **(B)** IL-17^+^ TNF-α^-^. **(C)** IL-17^+^ IFN-γ^+^. **(D)** IL-17^+^ IFN-γ^-^. **(E)** IL-17^+^ GM-CSF^+^. **(F)** IL-17^+^ GM-CSF^-^. **(G)** IL-17^+^ IL-22^+^. **(H)** IL-17^+^ IL-22^-^. CD4/CD8 ratios were calculated from absolute CD4 and CD8 counts obtained beyond the first 180 days (n = 32) post-infection. Measurements obtained after ART initiation or beyond 1-year post-infection were excluded. Correlations were assessed using Spearman rank correlation coefficient **(r**_**s**_), with linear regression lines shown for visualization purposes only. Two-tailed p-values are shown; statistical significance was defined as *p* < 0.05.

Total pre-HIV IL-17^+^ CD4^+^ T cells were inversely correlated with mean CD4/CD8 ratio within the first 180 days post-infection (r_s_ = -0.42, *p* = 0.012; Fig 1B) and ≥180 days post-infection (r_s_ = -0.55, *p* = 0.001; Fig 1C). A similar inverse relationship was observed for CD4/CD8 ratio at the last available measurement (Fig 1D and 1E). Within the first 180 days after infection, several IL-17-producing CD4^+^ T cell subsets including IL-17^+^ IFN-γ^+^, IL-17^+^ IFN-γ^-^, IL-17^+^ GM-CSF^+^, and IL-17^+^ IL-22^-^ populations, were significantly correlated with mean CD4/CD8 ratio (Fig 2). Beyond 180 days post-infection, most IL-17-producing CD4^+^ T cell subsets assessed remained significantly associated with mean CD4/CD8 ratio (Fig 3).

We also examined total IFN-γ^+^, GM-CSF^+^, and IL-22^+^ CD4^+^ T cells (S2 Fig) and found that these subsets were inversely correlated with mean CD4/CD8 ratio (S4 Fig).

### Pre-HIV IL-17^+^ CD4^+^ T cells are associated with faster CD4^+^ T cell decline after HIV infection

**Fig 4:**
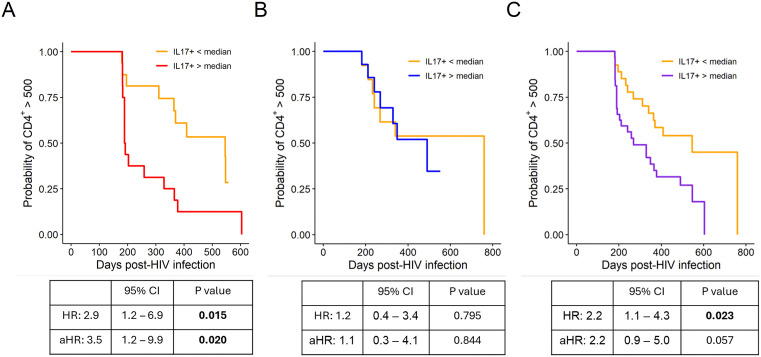
Total pre-HIV IL-17-producing CD4^+^ T cells were associated with faster CD4^+^ T cell decline in HVTN 503 but not PP/COS. PBMCs from study participants were stimulated ex vivo for 4 hours with PMA and ionomycin in the presence of Golgi Plug and Golgi Stop. The frequency of CD4^+^ T cells producing IL-17 was measured by flow cytometry. **(A)** Association in HVTN 503 (n = 32). **(B)** Association in PP/COS (n = 27). **(C)** Association in the combined cohort (n = 59). CD4 counts measured after ART initiation, after 1-year post-infection, or within the first 180 days post-infection were excluded. Hazard ratios (HR) were estimated using unadjusted Cox proportional hazards models. Multivariable models (adjusted HR[aHR]) were adjusted for sex, adenovirus type 5 (Ad5) titer, herpes simplex virus-2 (HSV-2) serostatus, age, and peak viral load for HVTN 503. For PP/COS and combined cohorts, models were adjusted for age, sex, HSV-2 serostatus, and peak viral load. Two-tailed p-values are shown; statistical significance was defined as *p* < 0.05.

We next examined the association between pre-HIV Th17 cells and CD4^+^ T cell decline. In the HVTN 503 cohort, higher frequencies of pre-HIV IL-17-producing CD4^+^ T cells were significantly associated with faster CD4 decline below 500 cells/mm^3^. Individuals with frequencies above the median experienced nearly a three-fold faster rate of CD4 decline in an unadjusted model (hazard ratio [HR] = 2.9, 95% confidence interval [CI]: 1.2 – 6.9, *p* = 0.015; Fig 4A). In a fully adjusted model including sex, adenovirus type 5 (Ad5) titer, herpes simplex virus-2 (HSV-2) serostatus, age, and peak viral load, pre-HIV IL-17-producing CD4^+^ T cells continued to be associated with faster CD4 decline (adjusted HR [aHR] = 3.5, 95% CI: 1.2 – 9.9, *p* = 0.020; Fig 4A). Given that viral load may mediate this relationship, we performed additional analyses adjusting for peak viral load alone (aHR = 2.5, 95% CI: 1.1 – 6.1, *p* = 0.038; S1 Table) and excluding peak viral load from the model (aHR = 4.3, 95% CI: 1.6 – 11.9, *p* = 0.005, S2 Table). In both cases, total pre-HIV IL-17-producing CD4^+^ T cells remained a significant predictor of faster CD4 decline. In contrast, no association was observed in the PP/COS cohort (HR = 1.2, 95% CI: 0.4 – 3.4, *p* = 0.795; Fig 4B). When data from both cohorts were combined, higher pre-HIV IL-17-producing CD4^+^ T cell frequencies were associated with faster CD4 decline in the unadjusted model (HR = 2.2, 95% CI: 1.1 – 4.3, *p* = 0.023) but did not reach statistical significance in the adjusted model (aHR = 2.2, 95% CI: 0.9 – 5.0, *p* = 0.057; Fig 4C). To assess whether this association extended to other cytokine-producing CD4^+^ T cell subsets, we examined total TNF-α, IFN-γ, GM-CSF, and IL-22 on CD4^+^ T cells. None of these subsets were associated with CD4 decline, indicating that the observed effect was specific to IL-17-producing CD4^+^ T cells (S3 Table).

Considering the disparity in associations between HVTN 503 and PP/COS, we investigated whether differences in immune activation (CD38^+^ HLA-DR^+^), HIV- co-receptor expression (CCR5^+^), or gut-homing (α4β7^hi^) CD4^+^ T cells could explain the observed cohort differences, as these subsets are known to be highly HIV-susceptible. We performed Spearman correlation analyses to examine the relationship between these cell subsets and total IL-17-producing CD4^+^ T cells (S5 Fig). Total pre-HIV IL-17^+^ CD4^+^ T cells showed no correlation with activated CD4^+^ T cells in either cohort, were only weakly correlated with α4β7^hi^ cells in PP/COS, but not in HVTN 503. Correlation with CCR5^+^ CD4^+^ T cells was modest but statistically significant in both cohorts. Together, these results suggest that the relationship with known HIV-susceptible subset cannot not explain the differences observed between the two cohorts.

### Interaction and stratified analyses

**Fig 5:**
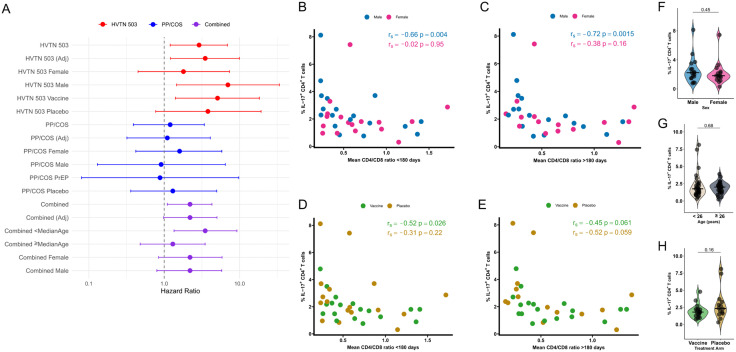
Stratified analyses of pre-HIV IL-17-producing CD4^+^ T cells and markers of HIV disease progression. PBMCs from study participants were stimulated ex vivo for 4 hours with PMA and ionomycin in the presence of Golgi Plug and Golgi Stop. The frequency of CD4^+^ T cells producing IL-17 was measured by flow cytometry. **(A)** Forest plot showing associations between total pre-HIV IL-17-producing CD4^+^ T cells and CD4 decline below 500 cells/mm^3^, stratified by sex, age, and treatment arm across cohorts. **(B, C)** Sex-stratified correlations between total pre-HIV IL-17-producing CD4^+^ T cell frequencies and mean CD4/CD8 ratio in HVTN 503. **(D, E)** Treatment arm-stratified correlations between total IL-17-producing CD4^+^ T cell frequencies and mean CD4/CD8 ratio in HVTN 503. **(F)** Comparison of total pre-HIV IL-17-producing CD4^+^ T cell frequencies stratified by sex in HVTN 503. **(G)** Comparison of total pre-HIV IL-17-producing CD4^+^ T cell frequencies stratified by age group in the combined cohort. **(H)** Comparison of total pre-HIV IL-17-producing CD4^+^ T cell frequencies stratified by treatment arm in HVTN 503. Hazard ratios were estimated using unadjusted Cox proportional hazards models. Multivariable models (Adj) were adjusted for sex, adenovirus type 5 (Ad5) titer, herpes simplex virus-2 (HSV-2) serostatus, age, and peak viral load for HVTN 503. For PP/COS and combined cohorts, models were adjusted for age, sex, HSV-2 serostatus, and peak viral load. CD4/CD8 ratios were calculated from absolute CD4 and CD8 counts obtained within the first 180 days or ≥180 days post-infection, as indicated. Measurements obtained after ART initiation or beyond 1-year post-infection were excluded. Correlations were assessed using Spearman rank correlation coefficient **(r**_**s**_). Group comparisons were performed using Mann-Whitney test. Two-tailed p-values are shown; statistical significance was defined as p < 0.05.

To further investigate cohort-specific differences and assess whether these associations extended across study population, we evaluated interactions between total pre-HIV IL-17-producing CD4^+^ T cells and selected variables, including age, sex, treatment arm, and viral load. No significant interactions were observed when cohorts were analyzed separately. However, in the combined analysis, a significant interaction was observed between IL-17-producing CD4^+^ T cells and age (*p* = 0.022). To further explore this, participants were stratified by age using the median cutoff (26 years). Among participants younger than 26 years, higher pre-HIV IL-17-producing CD4^+^ T cell frequencies were associated with faster CD4^+^ T cell decline (HR = 3.5; 95% CI: 1.35 – 9.22, *p* = 0.010; Fig 5A). In contrast, no significant association was observed among participants older than 26 years (HR = 1.3; 95% CI: 0.48 – 3.49, *p* = 0.615; Fig 5A). We next assessed the relationship between age and total IL-17-producing CD4^+^ T cells. No correlation was observed in HVTN 503 while a weak correlation was observed in PP/COS (S6 Fig). Notably, the distribution of age differed substantially between cohorts, with HVTN 503 participants restricted to 18 – 35 years, whereas PP/COS included participants spanning a broader age range, including individuals older than 35 years. To account for this imbalance, we repeated the analysis using an age cutoff of 35 years. The association between pre-HIV IL-17-producing CD4^+^ T cells and CD4 decline remained significant among participants younger than 35 years (HR = 2.8; 95% CI: 1.4 – 5.8); *p* = 0.005; S4 Table) but not among older participants (HR = 0.3; 95% CI: 0.03 – 3.6); *p* = 0.359; S4 Table). These findings suggest that differences in age distribution may partially explain cohort-specific associations observed.

In HVTN 503, sex-stratified analyses indicated that the association between pre-HIV IL-17-producing CD4^+^ T cells and CD4 decline was more pronounced in male participants (HR = 7.0, 95% CI: 1.46 – 33.7, *p* = 0.015; Fig 5A) than in female participants (HR = 1.8, 95% CI: 0.454 – 7.36, *p* = 0.396; Fig 5A). Consistently, an inverse correlation between pre-HIV IL-17-producing CD4^+^ T cell subsets and mean CD4/CD8 ratio was observed in male participants, but not in female participants (Figs 5B, 5C, S7 and S8).

Stratification by treatment arm showed a significant association in the vaccine arm (HR = 5.1, 95% CI: 1.4 – 18.3, *p* = 0.013; Fig 5A), but not in the placebo arm (HR = 3.8, 95% CI: 0.7 – 19.3, *p* = 0.101; Fig 5A) Analysis with mean CD4/CD8 ratio revealed heterogenous associations; total IL-17-producing CD4^+^ T cells significantly correlated with mean CD4/CD8 ratio in the vaccine arm, while specific subsets including IL-17^+^ IFN-γ^+^ and IL-17^+^ GM-CSF^+^ in the placebo arm (Figs 5D, S9 and S10).

To determine whether subgroup-specific associations reflected underlying differences in IL-17-production by CD4^+^ T cells, we compared pre-HIV IL-17-producing CD4^+^ T cell frequencies across sex, age, and treatment arm strata. No significant differences were observed (Fig 5F–5H) suggesting that the observed subgroup effects are unlikely driven by baseline differences in IL-17 production.

### Pre-HIV IL17-producing CD4^+^ T cells are not associated with viral load

**Fig 6:**
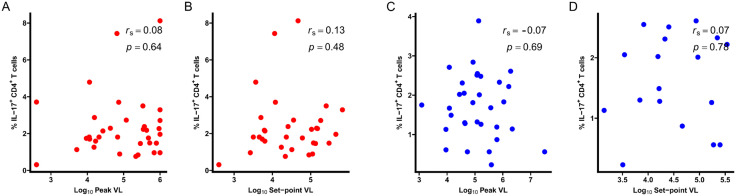
Pre-HIV IL-17-producing CD4^+^ T cells are not associated with viral load. **(A, B)** Correlation between pre-HIV IL-17-prooducing CD4^+^ T cells and peak (n = 35) and set-point (n = 32) viral load in HVTN 503. **(C, D)** Correlation between pre-HIV IL-17-prooducing CD4^+^ T cells and peak (n = 32) and set-point (n = 18) viral loads in PP/COS. Peak viral load was defined as the highest viral load measured within the first 180 days post-infection, while set-point viral load was defined as the mean viral load after 180 days post-infection. Viral load measurements obtained after ART initiation or beyond 1-year post-infection were excluded. Correlations were assessed using Spearman rank correlation coefficient **(r**_**s**_). Two-tailed p-values are shown; statistical significance was defined as *p* < 0.05.

We next assessed the relationship between pre-HIV IL-17-producing CD4^+^ T cell frequency and viral load measures, including peak and set-point viral loads. Pre-HIV IL-17-producing CD4^+^ T cell frequencies were not significantly associated with either peak or set-point viral load in either cohort (Figs 6, S11 and S12). These findings suggest that the association between pre-HIV IL-17-producing CD4^+^ T cells and disease progression is unlikely to be mediated by differences in early viral replication.

## Discussion

This study demonstrated that higher pre-HIV circulating IL-17-producing CD4^+^ T cells predict faster CD4^+^ T cell decline and lower CD4/CD8 ratios, particularly in the HVTN 503 cohort. Notably, pre-HIV Th17 frequency was not associated with peak or set-point viral loads, indicating that its association with disease progression is not mediated solely by viremia. These findings suggests that the pool of susceptible Th17 cells present prior to infection may accelerate early immune damage independently of viral load. This is consistent with prior studies showing that baseline immune composition, particularly the abundance of HIV target cells, can influence subsequent disease progression [1,4,5,7,28]. Several mechanisms may explain these associations. Th17 cells are highly permissive to HIV infection, and a higher pre-infection frequency of these cells may expand the pool of target cells available to the virus, thereby accelerating early CD4^+^ T cell loss [17,19,29,30]. In addition, a higher Th17 frequency may indicate an altered balance between Th17 cells and regulatory T cells (Tregs). This balance is important for maintaining immune homeostasis, with Th17 cells supporting mucosal defense and Tregs limiting excessive immune activation [19,31,32]. HIV infection further disrupts this balance, often leading to rapid Th17 loss and a relative increase in Treg frequencies. This shift can impair mucosal barrier integrity, promote microbial translocation, and amplify systemic immune activation [32,33]. Consequently, individuals with higher baseline Th17 levels may experience a more pronounced post-infection imbalance, accelerating CD4^+^ T cell decline. Higher circulating Th17 cells may also reflect increased baseline immune activation prior to infection or a greater proportion of mucosa-homing α4β7^+^ cells, which may support viral replication in gut-associated tissues [19,24,26].

A notable finding in our study is that this association was observed in the HVTN 503 cohort but not in the PP/COS cohort, suggesting the influence of cohort-specific modifiers. One possible explanation relates to differences in the composition of Th17 cells between cohorts. Th17 cells are heterogenous and include subsets that differ in expression of mucosal homing and HIV susceptibility markers such as α4β7 and CCR5 [19,26]. Cells co-expressing these markers are preferentially targeted and depleted during early HIV infection due to their enhanced permissiveness. In our prior work, IL-17^+^ CD4^+^ T cells showed a stronger correlation with α4β7^+^ CD4^+^ T cells in HVTN 503 compared to PP/COS [5], suggesting enrichment of mucosa-homing, highly susceptible subsets in this cohort. While this relationship was not evident in the current progression dataset, likely due to limited sample size, differences in Th17 cell composition, rather than overall frequency alone, may influence their impact on disease progression.

Age-related differences in immune function may also contribute to the observed cohort-specific effects. Ageing is associated with changes in CD4^+^ T cell composition, including reduced frequencies of memory Th17 cells and altered expression of chemokine receptors involved in mucosal trafficking [34]. In addition, older individuals often exhibit features of immunesenescence and chronic inflammation, which are linked to poorer clinical outcomes in HIV infection [35,36]. In our study, the association between pre-HIV Th17 frequency and CD4^+^ T cell decline was more pronounced in younger participants when cohorts were analyzed together, suggesting that Th17 cells may play a more prominent role in early disease progression in younger individuals. In contrast, in older individuals, other age-related factors may have greater influence on disease progression. This may partly explain the lack of association observed in the PP/COS cohort, which included relatively older participants. However, given the relatively narrow age range of our cohorts, these findings should be interpreted with caution and require confirmation in larger and more age-diverse populations.

Cohort-specific exposures may further influence the relationship between Th17 cells and HIV progression. HVTN 503 participants were enrolled in a trial evaluating an Ad5-vectored vaccine [27]. Although the vaccine did not confer protection, a previous study in healthy Ad5-seropositive individuals demonstrated that Ad5-specific immune stimulation preferentially expanded activated, mucosal-homing memory CD4^+^ T cells expressing α4β7 and CCR5, with increased susceptibility to HIV infection. [37]. Notably, these markers are highly co-expressed on IL-17-prdoucing CD4^+^ T cells. Such expansion of HIV-susceptible CD4^+^ T cells may increase the pool of target cells available for early infection, thereby amplifying viral replication and CD4^+^ T cell depletion. This could contribute to the stronger association between pre-HIV Th17 cells and disease progression observed in HVTN 503. In contrast, participants in the PP/COS cohort were exposed to oral pre-exposure prophylaxis (PrEP) [38], which has been reported to have minimal or transient effects on immune cell distribution [39,40]. While the immunological impact of PrEP in this context remains unclear, we speculate that differences in baseline immune environment between cohorts may have contributed to the different findings between cohorts.

In HVTN 503, male participants exhibited more pronounced associations between pre-HIV Th17 cell frequency and accelerated CD4 decline and lower CD4/CD8 ratios. Sex-based differences in HIV disease outcomes have been previously reported, with men showing lower baseline CD4 counts and distinct early immune responses compared to women during acute infection [41–44]. Sex bias in the Th17 pathway is also well documented [45–47]. Experimental studies have shown that androgen receptor signaling can modulate Th17 differentiation and IL-17 production, while estrogen signaling can promote Th17 responses, leading to sex-dependent differences in Th17 function [48–52]. These hormonally mediated differences may contribute to distinct immune set-points between men and women and provide a biological basis for the sex-specific associations observed in this study. Further studies are needed to clarify how sex-dependent regulation of Th17 cells influences early HIV pathogenesis.

Taken together, the association between pre-HIV Th17 cell frequency and disease progression appears context-dependent and likely reflects multifactorial influences, including cohort-specific exposures (such as Ad5 vaccination in HVTN 503), age-related differences, variation in Th17 cell composition, and biological sex. It should be highlighted that unmeasured factors such as circulating viral strains, host genetics, co-infection history, and other variables could drive cohort-specific findings. Overall, these observations highlight the importance of considering population context and cohort-specific biology when interpreting immunological predictors of HIV outcomes.

While our findings emphasize a role for circulating Th17 cells in HIV disease progression, several limitations should be considered. Our analysis focused on circulating Th17 cells, which may not fully reflect immune dynamics at mucosal sites where HIV replication predominantly occurs. Nonetheless, they may still provide insight into immune processes relevant to mucosal sites, supporting their use as a surrogate when mucosal sampling is not feasible. Future studies incorporating additional biological compartments, particularly mucosal tissues, are needed to extend these findings beyond circulating immune profiles. Our study may also be underpowered to detect interaction effects within individual cohorts, and confirmation of these findings will require larger studies. Missing CD8 data in one cohort limited comprehensive CD4/CD8 ratio analyses, and the geographic focus on African cohorts may limit generalizability. Finally, we were unable to assess how immune parameters change from pre- to post- infection, as post-infection PBMC samples for IL-17 and other functional measurements were not available. Understanding these dynamics, including HIV-specific responses and immune activation, represents an important direction for future studies.

## Conclusion

Elevated pre-HIV IL-17-producing CD4^+^ T cells were associated with faster HIV disease progression, independent of viral load, with effects that varied by cohort, age and sex. These findings highlight pre-infection Th17 cell abundance as a potential biomarker that warrants further validation for identifying individuals at higher risk of rapid disease progression and underscore its relevance to early HIV pathogenesis. Future studies are needed to investigate the mechanisms underlying these associations, including how age- and sex-related differences in immunity influence Th17 cell dynamics and HIV outcomes, and to explore whether interventions that modify Th17 cell function could slow disease progression.

## Methods

### Ethics statement

All participants in the parent cohorts provided informed written consent to have specimens stored for future immunological research, and the sub-studies reported here were approved by the institutional review board (IRB) at the University of Manitoba and at local IRBs where the study was conducted, where required.

### Study cohorts

Study participants were enrolled from two independent cohorts situated in South and East Africa, namely, HVTN 503 (N = 35) and Partners PrEP (N = 25), respectively. A subset of samples from Couples Observational Study (N = 7), another East African cohort similar to Partners PrEP, was included in our analyses. Hence, the renaming of our East African cohorts to PP/COS (N = 32). Details about each cohort and participants enrolment have been described elsewhere [27,38,53–55]. Study cohorts comprise of heterosexual, high-risk, HIV negative men and women above 18 years from multiple sites in South Africa (Cape Town, Soweto, MEDUNSA, eThekwini and Klerksdorp-Orkney-Stilfontein-Hartbeesfontein [KOSH]) for HVTN 503 and Kenya (Kisumu, Nairobi, Thika, Eldoret) and Uganda (Kampala, Jinja, Kabwohe, Mbale, Tororo) for PP/COS. Specifically, PP/COS comprised of couples in a serodiscordant relationship, where one partner is living with HIV and the other is not. PBMCs collected prior to HIV infection and cryopreserved were analyzed from participants who seroconverted during the original study follow-up.

### Sample processing

Cryopreserved PBMCs were thawed, washed and rested in warm media (RPMI 1640 supplemented with 10% fetal bovine serum and 1% Penicillin-Streptomycin) for 3 hours at 37^0^C and 5% CO_2_. Cell count and viability estimate were done manually using a hematocytometer. Cells were stimulated for 4 hours at 37^0^C with phorbol 12-myristate 13-acetate (PMA; 100 ng/ml) and ionomycin (1 μg/ml). Protein secretion was inhibited by the addition of pre-titrated amount of BD Golgi Stop (Monensin) and Golgi Plug (Brefeldin A) during stimulation.

### Intracellular cytokine staining and flow cytometry analyses

Following stimulation, cells were first stained with pre-titrated amount of LIVE/DEAD Fixable Aqua Dead Cell Stain Kit (Thermofisher Scientific) for 20 minutes to discriminate between live and dead cells. This was then followed by surface staining with a premixed cocktail of extracellular fluorochrome-conjugated monoclonal antibodies, including CD3-APCH7, CD4-BUV496, CD8-BV650, CD45RO-BUV395 and Integrin β7-BUV737, for 20 minutes at 4^0^C. Stained cells were washed, permeabilized using BD Cytofix/Cytoperm at 4^0^C for 20 minutes and stained with a premixed cocktail of intracellular cytokine antibodies, including IL-17A-BV421, IL-22-PE, GM-CSF-PECF594, IFN-γ-PECY7, TNF-α-FITC, IL-4-BV786 and IL-10-BV711, at 4^0^C for 30 minutes. Detailed information on reagents and flow cytometry panel of antibodies used for immunophenotyping are shown in S5–S7 Tables. Cells were then washed and acquired on a BD LSRFortessa Flow Cytometer. Immunophenotyping analysis was performed on FlowJo version 10.6.1 (BD Life Sciences). All experiments were performed in a blinded fashion.

### Th17 cell characterization

Th17 cells were defined by flow cytometry as IL-17^+^ CD4^+^ T cells. Additional cytokines produced by these cells, including IL-22, TNF- α, IFN- γ, and GM-CSF, were also measured, and corresponding subsets were gated in relation to IL-17A expression.

### Statistical analyses

Demographic data were summarized as median (interquartile ranges) and percentages for continuous and categorical variables, respectively. Pre-HIV infection samples collected within 1 year before the evidence of HIV infection were used to analyze the correlation between immune subsets and various parameter of HIV progression, including peak and set-point viral load, CD4/CD8 ratio within and beyond the initial 180 days post-infection and the decline in CD4^+^ T cell counts measured post-infection, before the initiation of ART. Spearman rank correlation test was used to evaluate the correlation with HIV peak and set-point viral loads. Peak viral load was defined at the highest viral load within the first 180 days post-infection prior to ART initiation, while set-point viral load was defined as the average viral load more than 180 days post-infection before ART initiation. For HVTN 503 only, Spearman correlation test was used to evaluate the correlation between IL-17-producing CD4^+^ T cell frequencies and mean CD4/CD8 ratio and CD4/CD8 ratio at the last available measurement post-HIV infection before ART initiation. Cox regression analysis was used to predict time to CD4^+^ T cell decline, with the end point defined as CD4^+^ T cell counts below 500 copies/ mm^3^ before ART initiation. The main exposure variable, pre-HIV IL-17^+^ CD4^+^ T cells frequency was dichotomized around the median expression level. CD4^+^ T cell counts measured during the first 180 days of infection were censored to exclude the transient CD4 count changes characteristic of early HIV infection [1]. CD4 counts measured after ART initiation were also excluded. All statistical analyses and plots were computed in RStudio 4.2.2. All statistics are two-tailed and p-values <0.05 were considered statistically significant.

## Supporting information

S1 Fig Representative bivariate flow cytometry plots illustrating gating strategy for IL-17-producing CD4^+^ T cells in relations to other cytokines. Other cytokines include IFN-γ, TNF-α, GM-CSF, and IL-22.
(TIF)

S2 Fig Representative flow cytometry plots illustrating the gating strategy for total IFN-γ^+^, GM-CSF^+^, TNF-α^+^, and IL-22^+^ CD4^+^ T cell cytokine subsets. (TIF)

S3 Fig Representative flow cytometry plots illustrating fluorescence minus one (FMO) control for measured CD4^+^ T cells cytokines. (TIF)

S4 Fig Correlation between mean CD4/CD8 ratio and other CD4^+^ T cell cytokine subsets in HVTN 503. PBMCs from study participants were stimulated ex vivo for 4 hours with PMA and ionomycin in the presence of Golgi Plug and Golgi Stop. The frequency of CD4^+^ T cells producing cytokines was measured by flow cytometry. < 180 days post-infection (n = 35). ≥ 180 days post-infection (n = 32). CD4/CD8 ratios were calculated from absolute CD4 and CD8 counts measured within and after the first 180 days post-infection. Measurements obtained after ART initiation or beyond 1-year post-infection were excluded. Correlations were assessed using Spearman rank correlation coefficient (r_s_), with linear regression lines shown for visualization purposes only. Two-tailed p-values are shown; statistical significance was defined as *p* < 0.05.
(TIF)

S5 Fig Correlation between total pre-HIV IL-17^+^ CD4^+^ T cells and α4β7^hi^, CCR5^+^, and CD38^+^ HLA-DR^+^ activated CD4^+^ T cells. (A – C) HVTN 503 (n = 35). (D – F) PP/COS (n = 32). Correlations were assessed using Spearman’s rank correlation coefficient (r_s_). Two-tailed p-values are shown; statistical significance was defined as p < 0.05.
(TIF)

S6 Fig Correlation between age and total pre-HIV IL-17-producing CD4^+^ T cells. (A) in HVTN 503. (B) in PP/COS. Correlations were assessed using Spearman’s rank correlation coefficient (r_s_). Two-tailed p-values are shown; statistical significance was defined as p < 0.05.
(TIF)

S7 Fig Sex-stratified correlation between mean CD4/CD8 ratio within the first 180 days post-infection and pre-HIV IL-17-producing CD4^+^ T cell subsets in HVTN 503. CD4/CD8 ratios were calculated from absolute CD4 and CD8 counts measured within the first 180 days post-infection (n = 35). Measurements obtained after ART initiation or beyond 1-year post-infection were excluded. Correlations were assessed using Spearman rank correlation coefficient (r_s_). Two-tailed p-values are shown; statistical significance was defined as *p* < 0.05.
(TIF)

S8 Fig Sex-stratified correlation between mean CD4/CD8 ratio ≥180 days post-infection and pre-HIV IL-17-producing CD4^+^ T cell subsets in HVTN 503. CD4/CD8 ratios were calculated from absolute CD4 and CD8 counts measured after the initial 180 days post-infection (n = 32). Measurements obtained after ART initiation or beyond 1-year post-infection were excluded. Correlations were assessed using Spearman rank correlation coefficient (r_s_). Two-tailed p-values are shown; statistical significance was defined as *p* < 0.05.
(TIF)

S9 Fig Treatment arm-stratified correlation between mean CD4/CD8 ratio within the first 180 days post-infection and pre-HIV IL-17-producing CD4^+^ T cell subsets in HVTN 503. CD4/CD8 ratios were calculated from absolute CD4 and CD8 counts measured within the first 180 days post-infection (n = 35). Measurements obtained after ART initiation or beyond 1-year post-infection were excluded. Correlations were assessed using Spearman rank correlation coefficient (r_s_). Two-tailed p-values are shown; statistical significance was defined as *p* < 0.05.
(TIF)

S10 Fig Treatment arm-stratified correlation between mean CD4/CD8 ratio ≥180 days post-infection and pre-HIV IL-17-producing CD4^+^ T cell subsets in HVTN 503. CD4/CD8 ratios were calculated from absolute CD4 and CD8 counts measured after the initial 180 days post-infection (n = 32). Measurements obtained after ART initiation or beyond 1-year post-infection were excluded. Correlations were assessed using Spearman rank correlation coefficient (r_s_). Two-tailed p-values are shown; statistical significance was defined as *p* < 0.05.
(TIF)

S11 Fig Correlation between peak viral load and pre-HIV IL-17-producing CD4^+^ T cell subsets. HVTN 503 (n = 35) and PP/COS (n = 32). Peak viral load was defined as the highest viral load measured within the first 180 days post-infection. Viral load measurements obtained after ART initiation or beyond 1-year post-infection were excluded. Correlations were assessed using Spearman rank correlation coefficient (r_s_). Two-tailed p-values are shown; statistical significance was defined as *p* < 0.05.
(TIF)

S12 Fig Correlation between set-point viral load and pre-HIV IL-17-producing CD4^+^ T cell subsets. HVTN 503 (n = 32) and PP/COS (n = 18). Set-point viral load was defined as the mean viral load measured after 180 days post-infection. Viral load measurements obtained after ART initiation or beyond 1-year post-infection were excluded. Correlations were assessed using Spearman rank correlation coefficient (r_s_). Two-tailed p-values are shown; statistical significance was defined as *p* < 0.05.
(TIF)

S1 Table Association between total pre-HIV IL-17^+^ CD4^+^ T cells and CD4 decline below 500 cells/mm^3^, adjusted for viral load (HVTN 503). Hazard ratios were estimated using Cox proportional hazards model. The model was adjusted only for peak viral load. Two-tailed p-values are shown; statistical significance was defined as *p* < 0.05. Abbreviations: aHR = Adjusted Hazard Ratio; CI = Confidence Interval; HVTN = HIV Vaccine Trials Network.
(PDF)

S2 Table Association between total pre-HIV IL-17^+^ CD4^+^ T cells and CD4 decline below 500 cells/mm^3^, adjusted for covariates excluding viral load (HVTN 503). Hazard ratios were estimated using Cox proportional hazards model. The model was adjusted for sex, adenovirus type 5 (Ad5) titer, herpes simplex virus-2 (HSV-2) status, and age. Two-tailed p-values are shown; statistical significance was defined as *p* < 0.05. Abbreviations: aHR = Adjusted Hazard Ratio; CI = Confidence Interval; HVTN = HIV Vaccine Trials Network.
(PDF)

S3 Table Association between other pre-HIV CD4^+^ T cell cytokine subsets and CD4 decline below 500 cells/mm^3^. Frequency of each phenotype below the median value was used as the reference category. Hazard ratios were estimated using unadjusted Cox proportional hazards model. Two-tailed p-values are shown; statistical significance was defined as *p* < 0.05. Abbreviations: HR = Hazard Ratio; CI = Confidence Interval; HVTN = HIV Vaccine Trials Network; PP/COS = Partners PrEP/Couples Observational Study.
(PDF)

S4 Table Association between total pre-HIV IL-17^+^ CD4^+^ T cells and CD4 decline below 500 cells/mm^3^, stratified by age cutoff at 35 years (combined cohorts). Hazard ratios were estimated using unadjusted Cox proportional hazards model. Two-tailed p-values are shown; statistical significance was defined as p < 0.05. Abbreviations: HR = Hazard Ratio; aHR = Adjusted Hazard Ratio; CI = Confidence Interval.
(PDF)

S5 Table Intracellular cytokine staining panel for ex vivo Th17 CD4^+^ T cell immunophenotyping in PBMC samples. (PDF)

S6 Table General reagents used for sample processing and data acquisition. (PDF)

S7 Table Stimulation reagents used for ex vivo Th17 immunophenotyping in PBMC samples. (PDF)
