# Supplementary figures and images for: Pre-Human Immunodeficiency Virus (HIV) infection Th17 CD4+ T cells as predictors of early HIV disease progression
Source: PLoS Pathog. 2026 Apr 24;22(4):e1013852. doi: 10.1371/journal.ppat.1013852 (PMC13132424; doi:10.1371/journal.ppat.1013852)

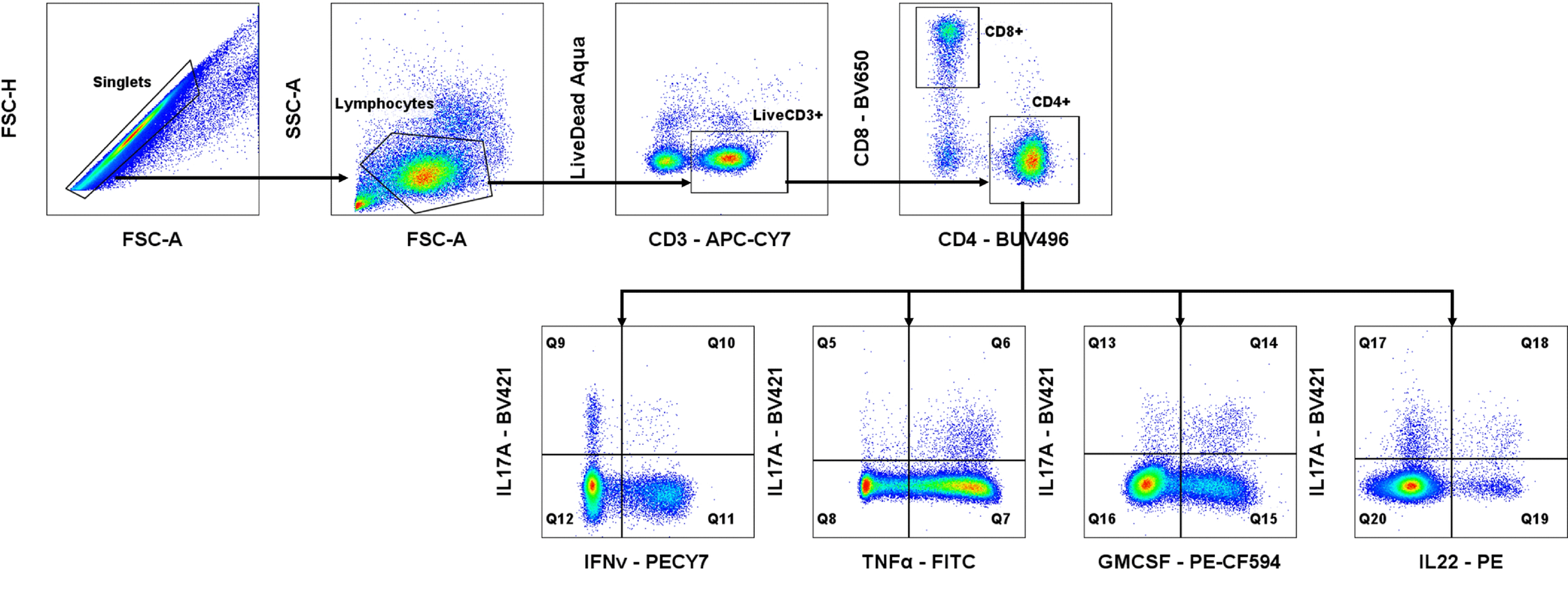

Supplement: S1 Fig — Other cytokines include IFN-γ, TNF-α, GM-CSF, and IL-22. (TIF) [file ppat.1013852.s001.tif]

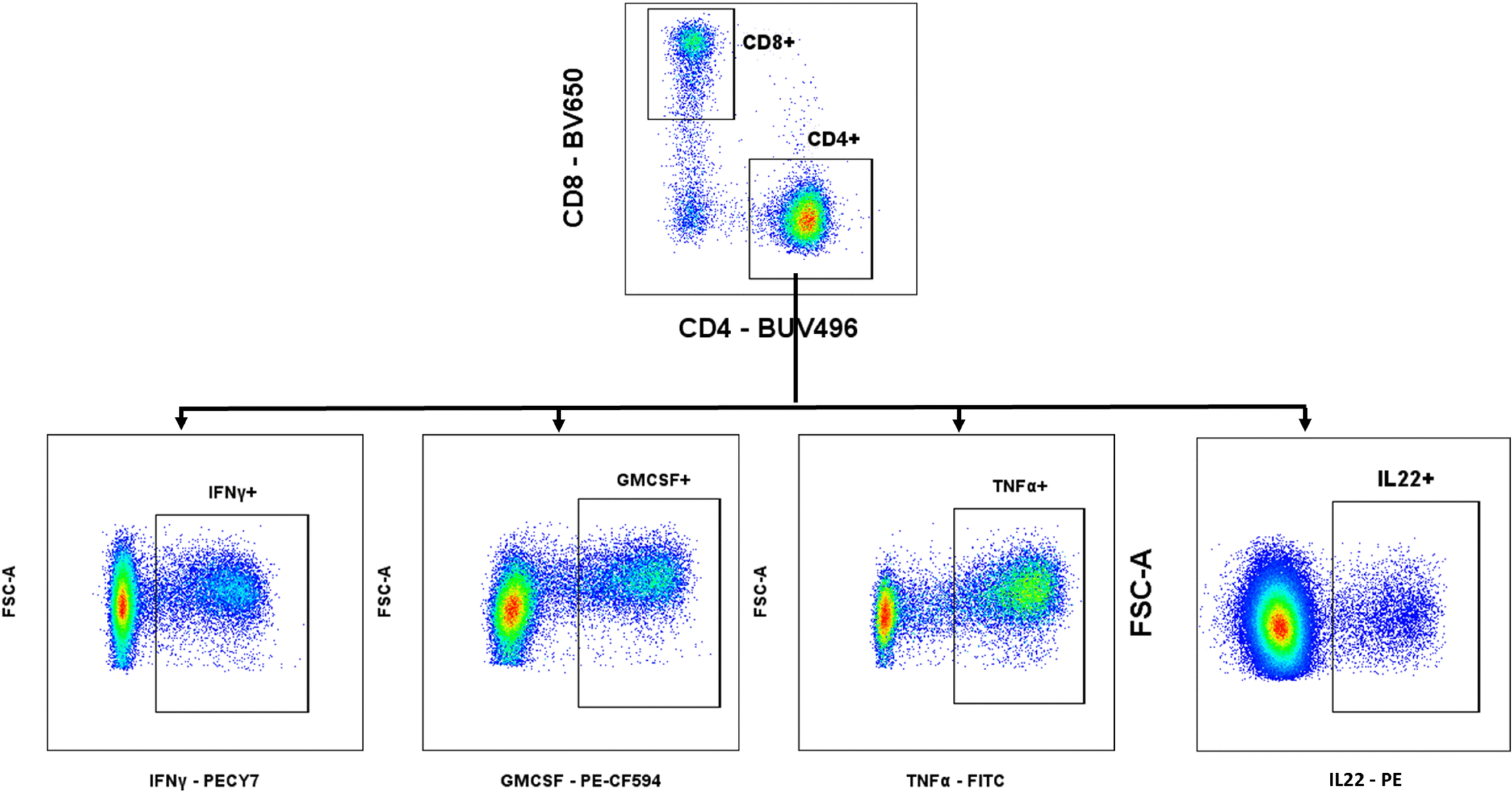

Supplement: S2 Fig — (TIF) [file ppat.1013852.s002.tif]

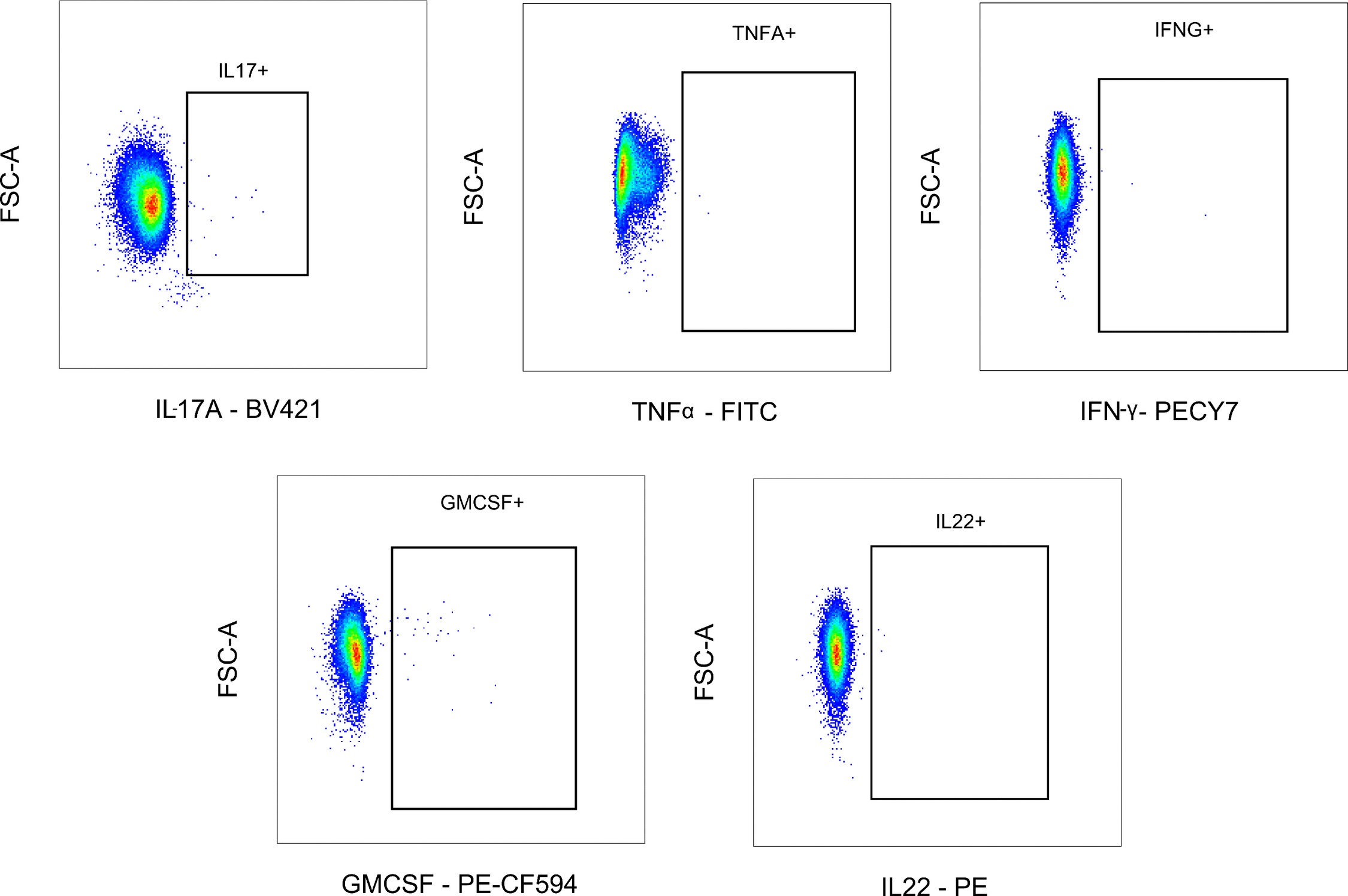

Supplement: S3 Fig — (TIF) [file ppat.1013852.s003.tif]

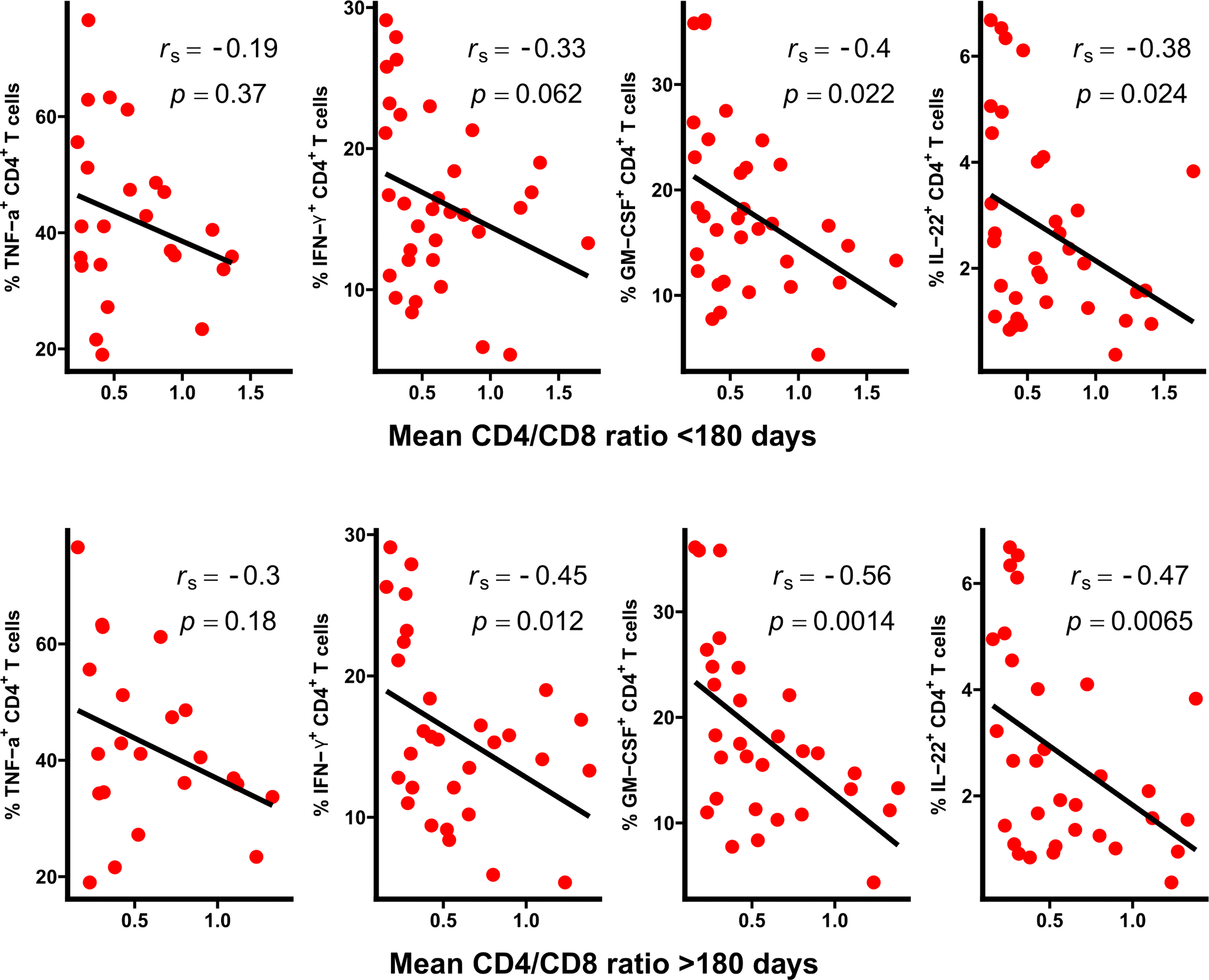

Supplement: S4 Fig — PBMCs from study participants were stimulated ex vivo for 4 hours with PMA and ionomycin in the presence of Golgi Plug and Golgi Stop. The frequency of CD4+ T cells producing cytokines was measured by flow cytometry. < 180 days post-infection (n = 35). ≥ 180 days post-infection (n = 32). CD4/CD8 ratios were calculated from absolute CD4 and CD8 counts measured within and after the first 180 days post-infection. Measurements obtained after ART initiation or beyond 1-year post-infection were excluded. Correlations were assessed using Spearman rank correlation coefficient (rs), with linear regression lines shown for visualization purposes only. Two-tailed p-values are shown; statistical significance was defined as p < 0.05. (TIF) [file ppat.1013852.s004.tif]

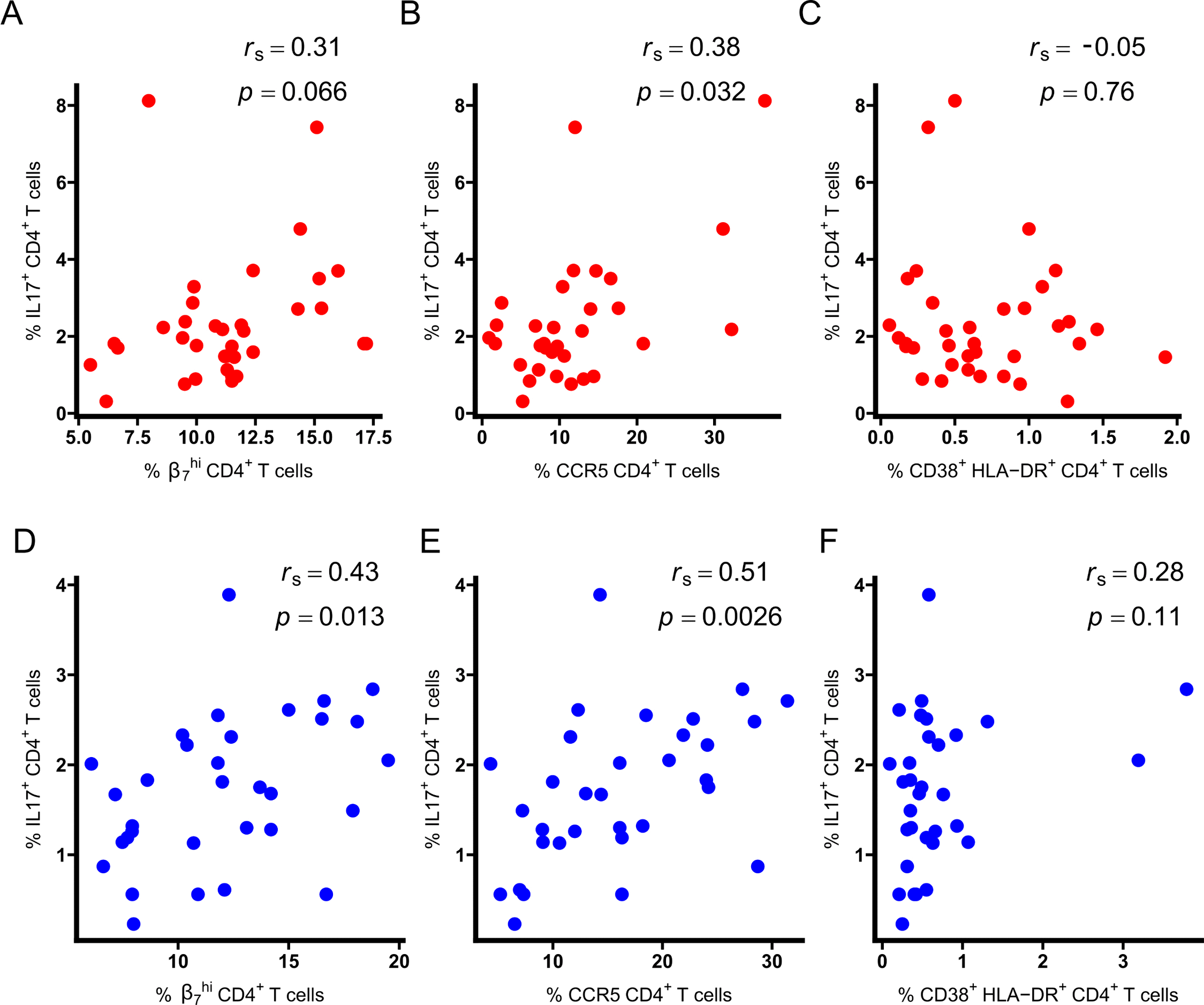

Supplement: S5 Fig — (A – C) HVTN 503 (n = 35). (D – F) PP/COS (n = 32). Correlations were assessed using Spearman’s rank correlation coefficient (rs). Two-tailed p-values are shown; statistical significance was defined as p < 0.05. (TIF) [file ppat.1013852.s005.tif]

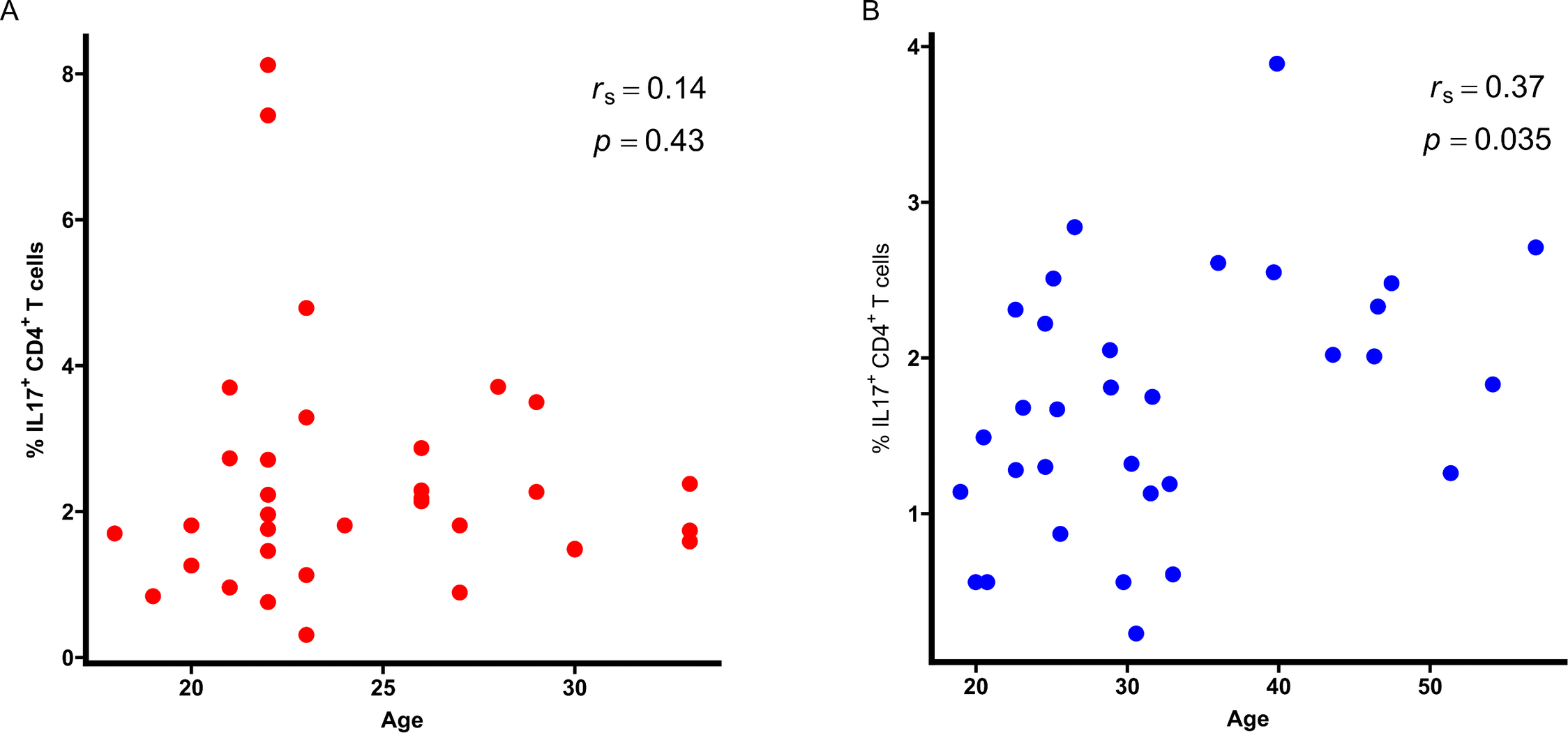

Supplement: S6 Fig — (A) in HVTN 503. (B) in PP/COS. Correlations were assessed using Spearman’s rank correlation coefficient (rs). Two-tailed p-values are shown; statistical significance was defined as p < 0.05. (TIF) [file ppat.1013852.s006.tif]

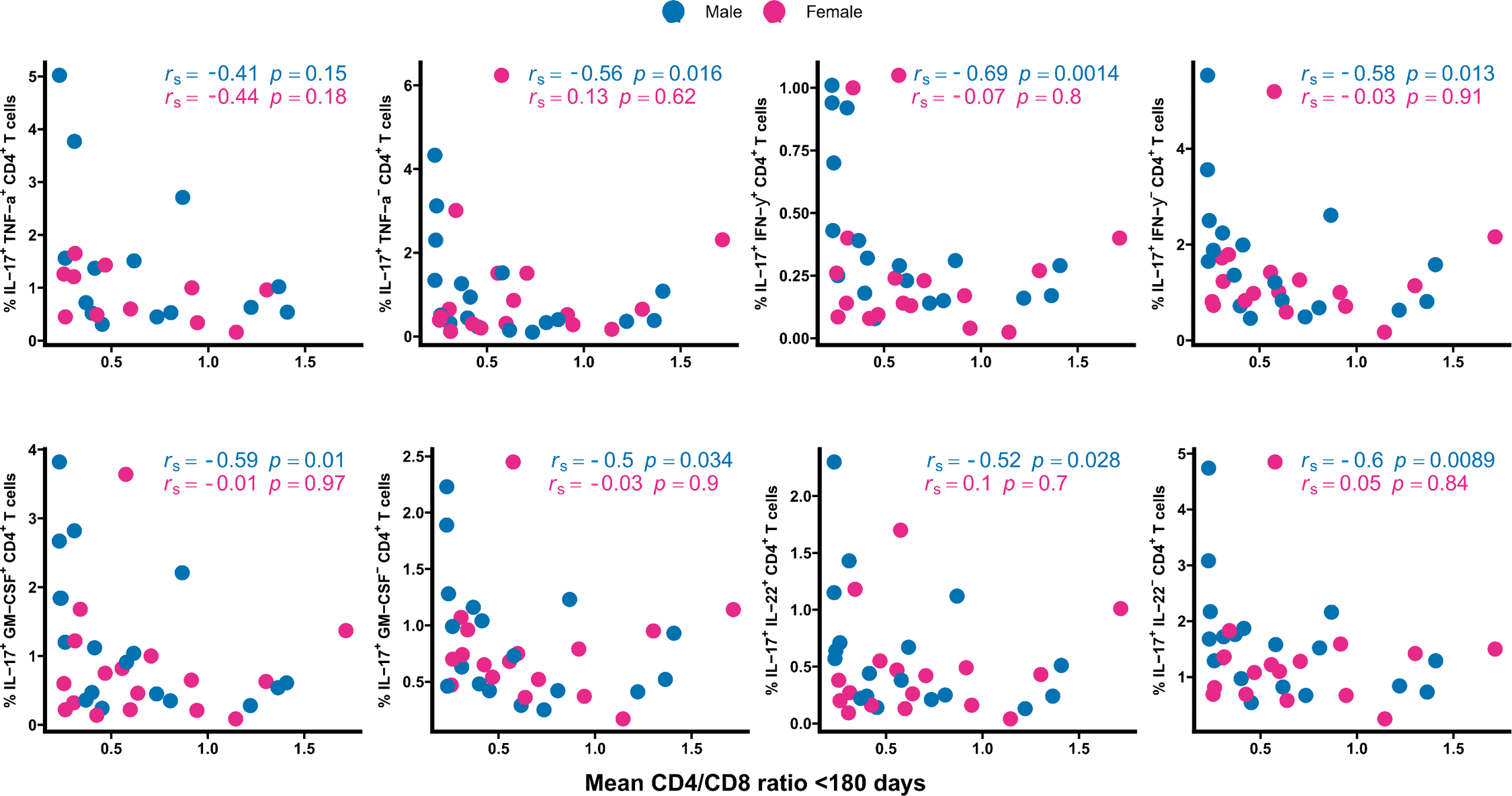

Supplement: S7 Fig — CD4/CD8 ratios were calculated from absolute CD4 and CD8 counts measured within the first 180 days post-infection (n = 35). Measurements obtained after ART initiation or beyond 1-year post-infection were excluded. Correlations were assessed using Spearman rank correlation coefficient (rs). Two-tailed p-values are shown; statistical significance was defined as p < 0.05. (TIF) [file ppat.1013852.s007.tif]

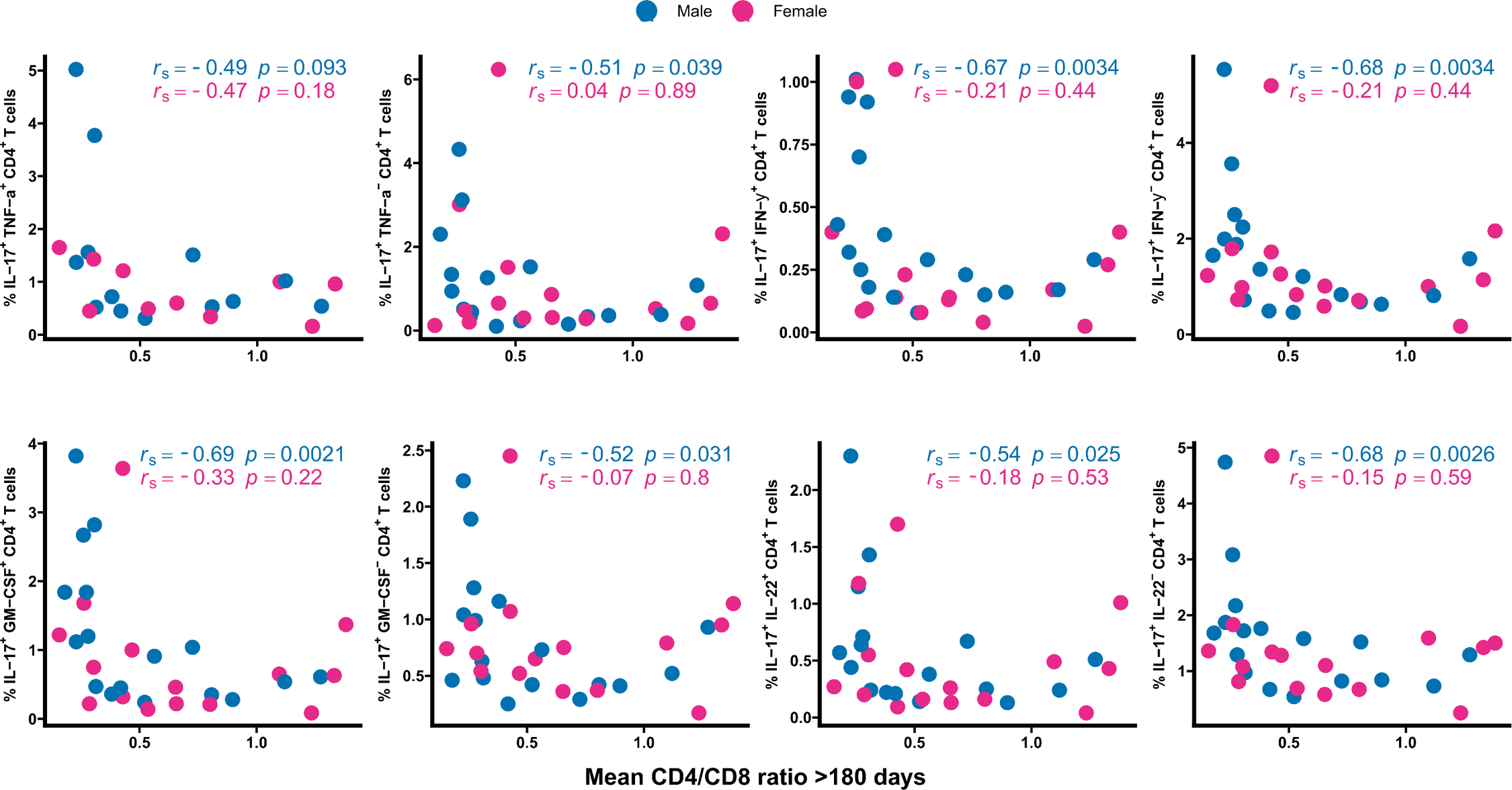

Supplement: S8 Fig — CD4/CD8 ratios were calculated from absolute CD4 and CD8 counts measured after the initial 180 days post-infection (n = 32). Measurements obtained after ART initiation or beyond 1-year post-infection were excluded. Correlations were assessed using Spearman rank correlation coefficient (rs). Two-tailed p-values are shown; statistical significance was defined as p < 0.05. (TIF) [file ppat.1013852.s008.tif]

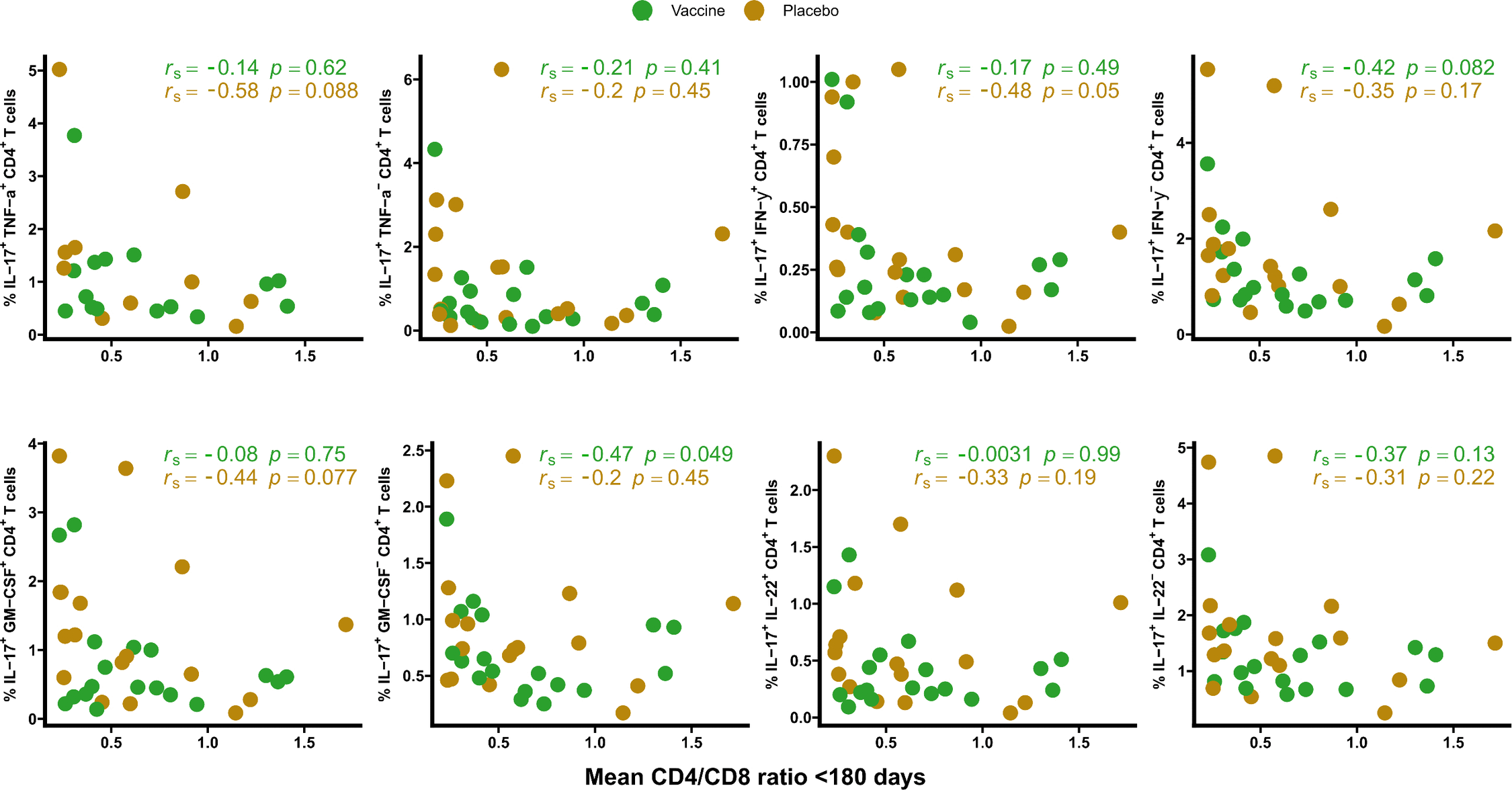

Supplement: S9 Fig — CD4/CD8 ratios were calculated from absolute CD4 and CD8 counts measured within the first 180 days post-infection (n = 35). Measurements obtained after ART initiation or beyond 1-year post-infection were excluded. Correlations were assessed using Spearman rank correlation coefficient (rs). Two-tailed p-values are shown; statistical significance was defined as p < 0.05. (TIF) [file ppat.1013852.s009.tif]

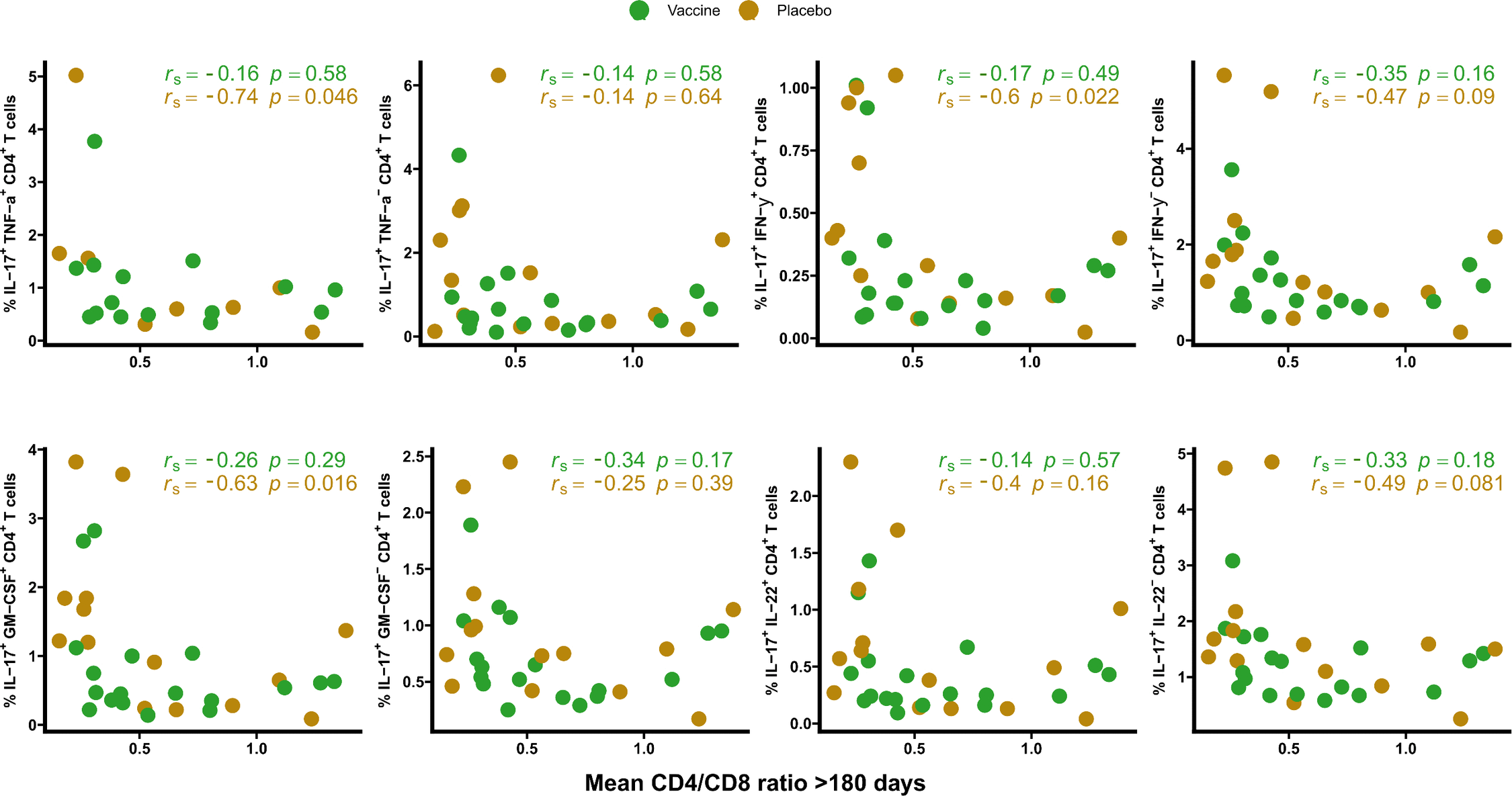

Supplement: S10 Fig — CD4/CD8 ratios were calculated from absolute CD4 and CD8 counts measured after the initial 180 days post-infection (n = 32). Measurements obtained after ART initiation or beyond 1-year post-infection were excluded. Correlations were assessed using Spearman rank correlation coefficient (rs). Two-tailed p-values are shown; statistical significance was defined as p < 0.05. (TIF) [file ppat.1013852.s010.tif]

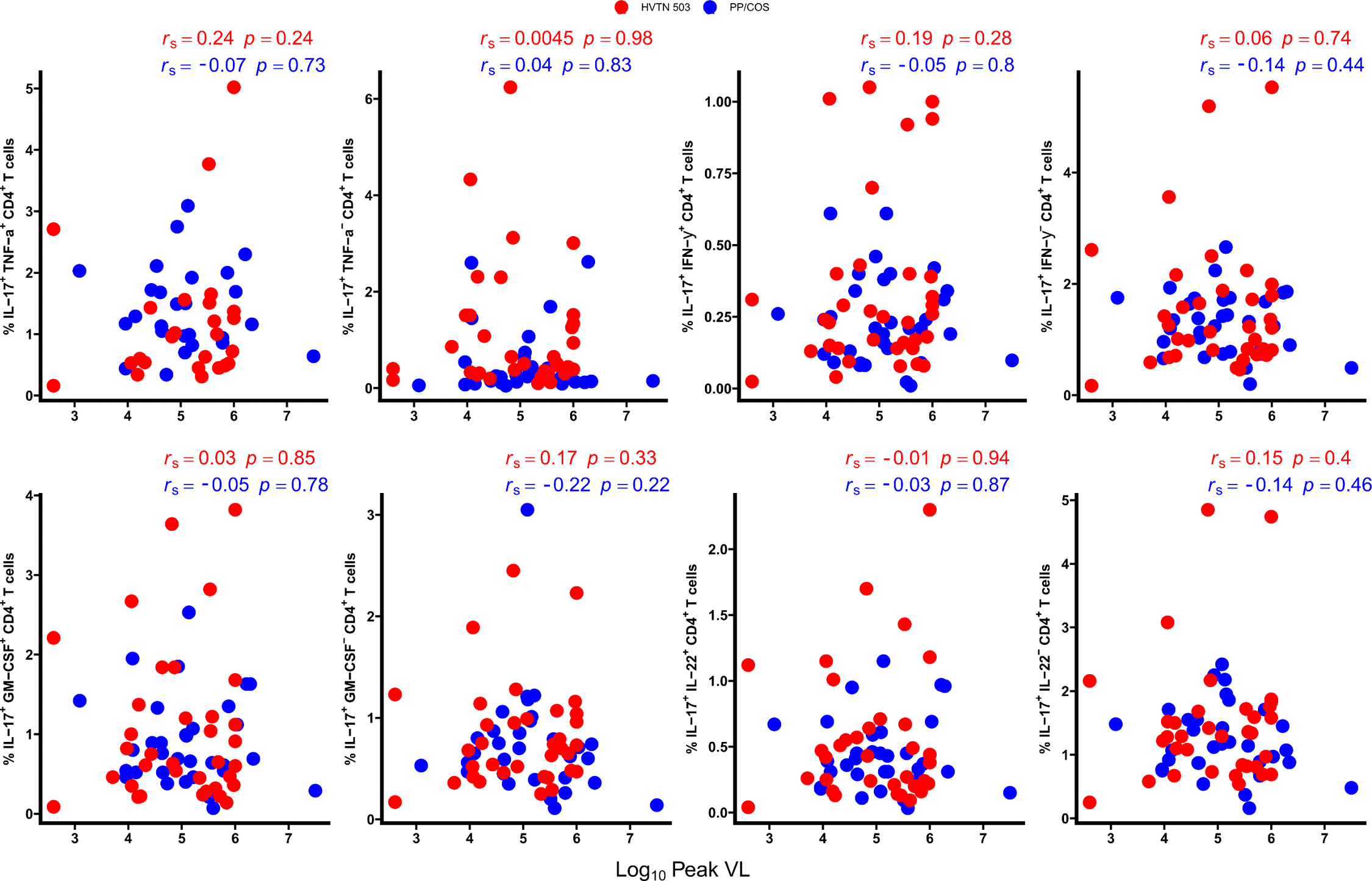

Supplement: S11 Fig — HVTN 503 (n = 35) and PP/COS (n = 32). Peak viral load was defined as the highest viral load measured within the first 180 days post-infection. Viral load measurements obtained after ART initiation or beyond 1-year post-infection were excluded. Correlations were assessed using Spearman rank correlation coefficient (rs). Two-tailed p-values are shown; statistical significance was defined as p < 0.05. (TIF) [file ppat.1013852.s011.tif]

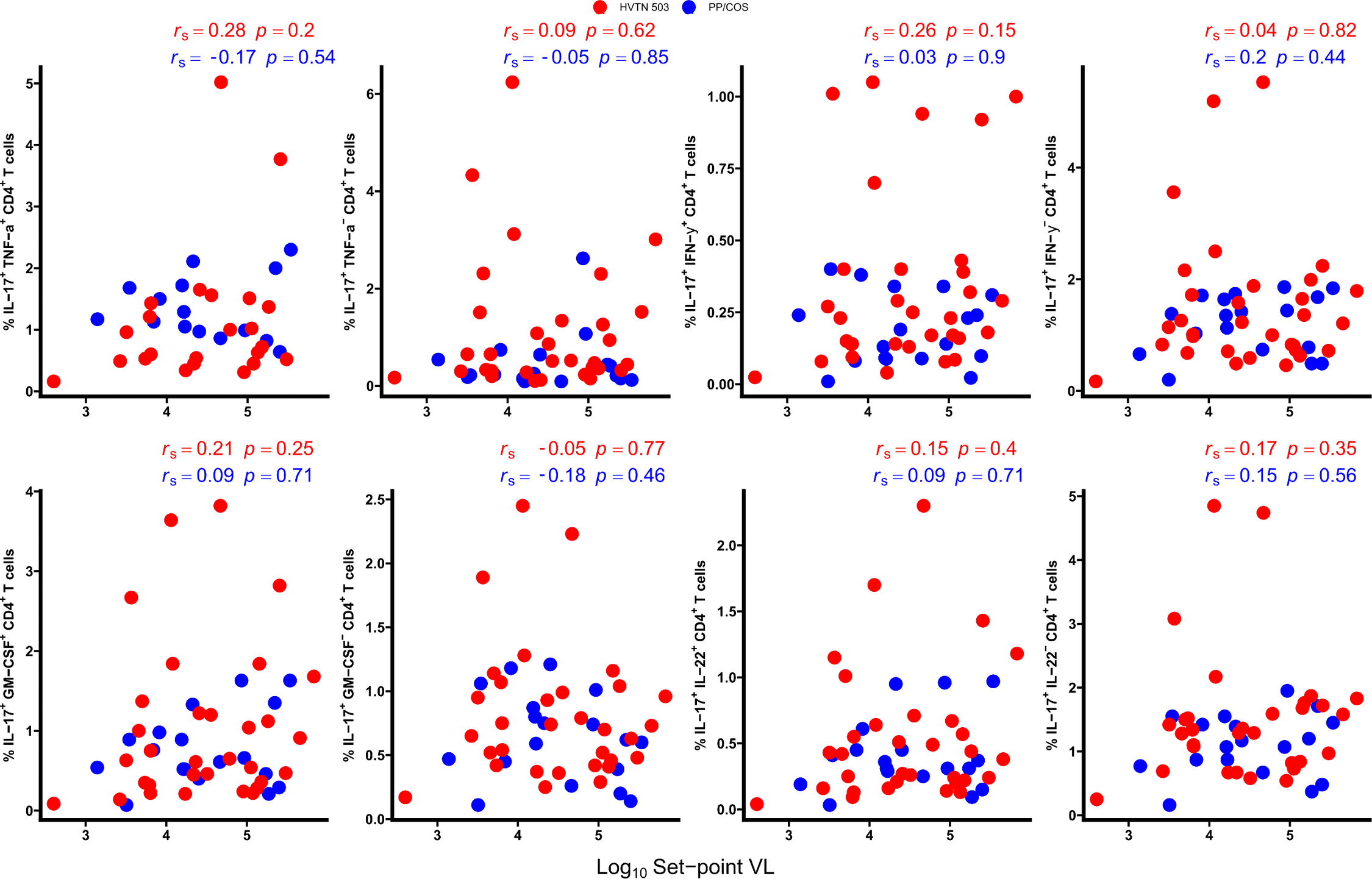

Supplement: S12 Fig — HVTN 503 (n = 32) and PP/COS (n = 18). Set-point viral load was defined as the mean viral load measured after 180 days post-infection. Viral load measurements obtained after ART initiation or beyond 1-year post-infection were excluded. Correlations were assessed using Spearman rank correlation coefficient (rs). Two-tailed p-values are shown; statistical significance was defined as p < 0.05. (TIF) [file ppat.1013852.s012.tif]
