# Supplementary material for: Pre-Human Immunodeficiency Virus (HIV) infection Th17 CD4+ T cells as predictors of early HIV disease progression
Source: PLoS Pathog. 2026 Apr 24;22(4):e1013852. doi: 10.1371/journal.ppat.1013852 (PMC13132424; doi:10.1371/journal.ppat.1013852)
Supplement: S1 Table — Hazard ratios were estimated using Cox proportional hazards model. The model was adjusted only for peak viral load. Two-tailed p-values are shown; statistical significance was defined as p < 0.05. Abbreviations: aHR = Adjusted Hazard Ratio; CI = Confidence Interval; HVTN = HIV Vaccine Trials Network. (PDF) [file ppat.1013852.s013.pdf]

S1 Table. Association between pre-HIV IL-17^{+} CD4^{+} T cells and CD4 decline below 500 cells/mm^{3}, adjusted for viral load (HVTN 503)

Variables	Units	aHR (95% CI)	*p* value
**IL17^{+}CD4^{+} T cells**	IL17^{+} < median	Ref	
	IL17^{+} > median	2.54 (1.05 – 6.12)	**0.038**
**Peak viral load**		1.62 (0.86 – 3.04)	0.135

Hazard ratios were estimated using Cox proportional hazards model. The model was adjusted only for peak viral load. Two-tailed p-values are shown; statistical significance was defined as $p < 0.05$. Abbreviations: aHR = Adjusted Hazard Ratio; CI = Confidence Interval; HVTN = HIV Vaccine Trials Network.
