# Supplementary material for: Pre-Human Immunodeficiency Virus (HIV) infection Th17 CD4+ T cells as predictors of early HIV disease progression
Source: PLoS Pathog. 2026 Apr 24;22(4):e1013852. doi: 10.1371/journal.ppat.1013852 (PMC13132424; doi:10.1371/journal.ppat.1013852)
Supplement: S2 Table — Hazard ratios were estimated using Cox proportional hazards model. The model was adjusted for sex, adenovirus type 5 (Ad5) titer, herpes simplex virus-2 (HSV-2) status, and age. Two-tailed p-values are shown; statistical significance was defined as p < 0.05. Abbreviations: aHR = Adjusted Hazard Ratio; CI = Confidence Interval; HVTN = HIV Vaccine Trials Network. (PDF) [file ppat.1013852.s014.pdf]

S2 Table. Association between pre-HIV IL-17$^+$ CD4$^+$ T cells and CD4 decline below 500 cells/mm^3, adjusted for covariates excluding viral load (HVTN 503)

Variables	Units	aHR (95% CI)	*p* value
**IL17$^+$CD4$^+$ T cells**	IL17$^+$ < median	Ref	
	IL17$^+$ > median	4.31 (1.57 – 11.85)	**0.005**

Hazard ratios were estimated using Cox proportional hazards model. The model was adjusted for sex, adenovirus type 5 (Ad5) titer, herpes simplex virus-2 (HSV-2) status, and age. Two-tailed p-values are shown; statistical significance was defined as $p < 0.05$. Abbreviations: aHR = Adjusted Hazard Ratio; CI = Confidence Interval; HVTN = HIV Vaccine Trials Network.
