# Supplementary material for: Pre-Human Immunodeficiency Virus (HIV) infection Th17 CD4+ T cells as predictors of early HIV disease progression
Source: PLoS Pathog. 2026 Apr 24;22(4):e1013852. doi: 10.1371/journal.ppat.1013852 (PMC13132424; doi:10.1371/journal.ppat.1013852)
Supplement: S3 Table — Frequency of each phenotype below the median value was used as the reference category. Hazard ratios were estimated using unadjusted Cox proportional hazards model. Two-tailed p-values are shown; statistical significance was defined as p < 0.05. Abbreviations: HR = Hazard Ratio; CI = Confidence Interval; HVTN = HIV Vaccine Trials Network; PP/COS = Partners PrEP/Couples Observational Study. (PDF) [file ppat.1013852.s015.pdf]

S3 Table. Association between other pre-HIV $CD4^+$ T cell cytokine subsets and CD4 decline below 500 cells/mm^3

Cohort	Variables	HR (95% CI)	*p* value
**HVTN 503**	TNF-α^+ $CD4^+$ T cells	1.40 (0.48 – 4.05)	0.533
	IFN-γ^+ $CD4^+$ T cells	1.68 (0.70 – 4.04)	0.242
	GM-CSF^+ $CD4^+$ T cells	1.35 (0.57 – 3.21)	0.498
	IL-22^+ $CD4^+$ T cells	1.38 (0.61 – 3.15)	0.440
**PP/COS**	TNF-α^+ $CD4^+$ T cells	1.1 (0.36 – 3.50)	0.837
	IFN-γ^+ $CD4^+$ T cells	2.3 (0.73 – 7.29)	0.153
	GM-CSF^+ $CD4^+$ T cells	0.79 (0.27 – 2.37)	0.677
	IL-22^+ $CD4^+$ T cells	1.1 (0.38 – 3.38)	0.821
**Combined**	TNF-α^+ $CD4^+$ T cells	0.52 (0.24 – 1.15)	0.106
	IFN-γ^+ $CD4^+$ T cells	1.1 (0.57 – 2.18)	0.758
	GM-CSF^+ $CD4^+$ T cells	1.4 (0.74 – 2.83)	0.286
	IL-22^+ $CD4^+$ T cells	1.4 (0.72 – 2.64)	0.331

Frequency of each phenotype below the median value was used as the reference category. Hazard ratios were estimated using unadjusted Cox proportional hazards model. Two-tailed p-values are shown; statistical significance was defined as $p < 0.05$. Abbreviations: HR = Hazard Ratio; CI = Confidence Interval; HVTN = HIV Vaccine Trials Network; PP/COS = Partners PrEP/Couples Observational Study.
