# Supplementary material for: Pre-Human Immunodeficiency Virus (HIV) infection Th17 CD4+ T cells as predictors of early HIV disease progression
Source: PLoS Pathog. 2026 Apr 24;22(4):e1013852. doi: 10.1371/journal.ppat.1013852 (PMC13132424; doi:10.1371/journal.ppat.1013852)
Supplement: S4 Table — Hazard ratios were estimated using unadjusted Cox proportional hazards model. Two-tailed p-values are shown; statistical significance was defined as p < 0.05. Abbreviations: HR = Hazard Ratio; aHR = Adjusted Hazard Ratio; CI = Confidence Interval. (PDF) [file ppat.1013852.s016.pdf]

S4 Table. Association between pre-HIV IL-17$^+$ CD4$^+$ T cells and CD4 decline below 500 cells/mm^3, stratified by age cutoff at 35 years (combined cohorts)

Cohort	< 35 Years		≥ 35 Years	
	**HR (95% CI)**	***P* value**	**HR (95% CI)**	***P* value**
**IL17$^+$ CD4$^+$ T cells**	2.8 (1.37 – 5.83)	**0.005**	0.32 (0.03 – 3.61)	0.359

Hazard ratios were estimated using unadjusted Cox proportional hazards model. Two-tailed p-values are shown; statistical significance was defined as $p < 0.05$. Abbreviations: HR = Hazard Ratio; aHR = Adjusted Hazard Ratio; CI = Confidence Interval.
