# Supplementary material for: Pre-Human Immunodeficiency Virus (HIV) infection Th17 CD4+ T cells as predictors of early HIV disease progression
Source: PLoS Pathog. 2026 Apr 24;22(4):e1013852. doi: 10.1371/journal.ppat.1013852 (PMC13132424; doi:10.1371/journal.ppat.1013852)
Supplement: S5 Table — (PDF) [file ppat.1013852.s017.pdf]

S5 Table. Intracellular cytokine staining panel for ex vivo Th17 $CD4^+$ T cell immunophenotyping in PBMC samples

Antibody	Fluorochrome	Titrated Volume/ dilution (µl)	Clone	Catalog Number	Company
**TNF-α**	FITC	4	MAb11	11-7349-41	Thermofisher Scientific
**IFN-γ**	PECy7	1	B27	557643	BD Biosciences
**GM-CSF**	PE-CF594	1	BVD2-21C11	562857	BD Biosciences
**IL-22**	PE	2	22URTI	12-7229-42	Thermofisher Scientific
**IL-17A**	BV421	4	N49-653	562933	BD Biosciences
**Live/Dead Aqua**	V500	1:500	Not Applicable	L34957	Thermofisher Scientific
**CD8**	BV650	0.5	SK1	344730	BioLegend
**IL-10**	BV711	4	JES3-9D7	564050	BD Biosciences
**IL-4**	BV786	4	MP4-25D2	564113	BD Biosciences
**CD3**	APC-H7	1	SK7	560176	BD Biosciences
**CD45RO**	BUV395	2	UCHL1	564291	BD Biosciences
**CD4**	BUV496	1	SK3	564651	BD Biosciences
**INTβ7**	BUV737	0.5	FIB504	565604	BD Biosciences
