# Supplementary material for: Pre-Human Immunodeficiency Virus (HIV) infection Th17 CD4+ T cells as predictors of early HIV disease progression
Source: PLoS Pathog. 2026 Apr 24;22(4):e1013852. doi: 10.1371/journal.ppat.1013852 (PMC13132424; doi:10.1371/journal.ppat.1013852)
Supplement: S6 Table — (PDF) [file ppat.1013852.s018.pdf]

S6 Table. General reagents used for sample processing and data acquisition

Reagents	Catalog Number	Company
**BD Cytofix/Cytoperm**	554722	BD Biosciences
**BD Perm Wash**	554723	BD Biosciences
**Brilliant Stain Buffer**	563794	BD Biosciences
**BD Cytometer Setup and Tracking (CS&T) beads**		BD Biosciences
**Rainbow Calibration Particles, 8 peaks (3·0 – 3·4µm)**	422903	BioLegend
**Anti-rat and anti-hamster Ig, k/Negative control Compensation particles set**	552845	BD Biosciences
**Anti-Mouse Ig, k/Negative control Compensation particles set**	552843	BD Biosciences
**LIVE/DEAD™ Fixable Aqua Dead Cell Stain Kit, for 405 nm excitation**	L34957	Thermofisher Scientific
**Arc Amine Reactive Compensation Bead Kit**	A10346	Thermofisher Scientific
**Roswell Park Memorial Institute (RPMI)-1640**	11875093	Thermofisher Scientific
**Gibco™ Phosphate Buffered Saline (PBS)**	10010023	Thermofisher Scientific
**Fetal Bovine Serum (FBS)**	MT35077CV	Thermofisher Scientific
**Dimethyl Sulfoxide (DMSO)**	BP231100	Thermofisher Scientific
**Penicillin-Streptomycin (PS)**	450-201-EL	Wisent
**Trypan Blue solution**	609-130-EL	Wisent
