# Supplementary material for: Pre-Human Immunodeficiency Virus (HIV) infection Th17 CD4+ T cells as predictors of early HIV disease progression
Source: PLoS Pathog. 2026 Apr 24;22(4):e1013852. doi: 10.1371/journal.ppat.1013852 (PMC13132424; doi:10.1371/journal.ppat.1013852)
Supplement: S7 Table — (PDF) [file ppat.1013852.s019.pdf]

S7 Table. Stimulation reagents used for ex vivo Th17 immunophenotyping in PBMC samples

Reagents	Catalog Number	Company
**Phorbol 12-myristate 13-acetate (PMA)**	P8139	Sigma-Aldrich
**Ionomycin**	I0634	Sigma-Aldrich
**BD GolgiPlug™ Protein Transport Inhibitor (Containing Brefeldin A)**	555029	BD Biosciences
**BD GolgiStop™ Protein Transport Inhibitor (Containing Monensin)**	554724	BD Biosciences
